# Estimating Risk Factor Time Paths Among People with Type 2 Diabetes and QALY Gains from Risk Factor Management

**DOI:** 10.1007/s40273-024-01398-4

**Published:** 2024-06-26

**Authors:** Ni Gao, Helen A. Dakin, Rury R. Holman, Lee-Ling Lim, José Leal, Philip Clarke

**Affiliations:** 1 Health Economics Research Centre, Nuffield Department of Population Health, University of Oxford, Old Road Campus, Headington, Oxford, OX3 7LF UK; 2 Centre for Health Economics, University of York, York, UK; 3 Diabetes Trials Unit, Radcliffe Department of Medicine, University of Oxford, Oxford, UK; 4 Department of Medicine, Faculty of Medicine, University of Malaya, Kuala Lumpur, Malaysia; 5 Department of Medicine and Therapeutics, The Chinese University of Hong Kong, Hong Kong, SAR China; 6 Asia Diabetes Foundation, Hong Kong, SAR China

## Abstract

**Objectives:**

Most type 2 diabetes simulation models utilise equations mapping out lifetime trajectories of risk factors [e.g. glycated haemoglobin (HbA_1c_)]. Existing equations, using historic data or assuming constant risk factors, frequently underestimate or overestimate complication rates. Updated risk factor time path equations are needed for simulation models to more accurately predict complication rates.

**Aims:**

(1) Update United Kingdom Prospective Diabetes Study Outcomes Model (UKPDS-OM2) risk factor time path equations; (2) compare quality-adjusted life-years (QALYs) using original and updated equations; and (3) compare QALY gains for reference case simulations using different risk factor equations.

**Methods:**

Using pooled contemporary data from two randomised trials EXSCEL and TECOS (*n* = 28,608), we estimated: dynamic panel models of seven continuous risk factors (high-density lipoprotein cholesterol, low density lipoprotein cholesterol, HbA_1c_, haemoglobin, heart rate, blood pressure and body mass index); two-step models of estimated glomerular filtration rate; and survival analyses of peripheral arterial disease, atrial fibrillation and albuminuria. UKPDS-OM2-derived lifetime QALYs were extrapolated over 70 years using historical and the new risk factor equations.

**Results:**

All new risk factor equation predictions were within 95% confidence intervals of observed values, displaying good agreement between observed and estimated values. Historical risk factor time path equations predicted trial participants would accrue 9.84 QALYs, increasing to 10.98 QALYs using contemporary equations.

**Discussion:**

Incorporating updated risk factor time path equations into diabetes simulation models could give more accurate predictions of long-term health, costs, QALYs and cost-effectiveness estimates, as well as a more precise understanding of the impact of diabetes on patients’ health, expenditure and quality of life.

**Trial Registration:**

ClinicalTrials.gov NCT01144338 and NCT00790205

**Supplementary Information:**

The online version contains supplementary material available at 10.1007/s40273-024-01398-4.

## Key Points for Decision Makers

**Table:** 

We present updated time paths for 11 diabetes risk factors using data on more than 80,000 patient-years of follow up from contemporary multinational clinical outcome trials.
Our results suggest that improvements in risk factor management over the last two decades have gained the average person with type 2 diabetes 1.13 QALYs.
These updated time path equations can be used in diabetes simulation models to better inform economic evaluations and health technology assessments.

## Introduction

Health economic computer simulation models are now widely used to project health outcomes and costs among individuals with type 2 diabetes and to inform the cost-effectiveness of novel interventions [1]. Such models are used to predict a variety of diabetes-related complications and death to estimate outcomes, such as quality-adjusted life years (QALYs), life expectancy and lifetime costs [2]. One of the most widely used diabetes simulation models is the United Kingdom Prospective Diabetes Study Outcomes Model (UKPDS-OM) [3]. UKPDS-OM is a multi-application model that has been used in a wide variety of applications, including cost-effectiveness analyses [4–6] and prediction of life expectancy [7, 8], as well as informing diabetes guidelines of health technology assessment organisations, such as NICE [9]. In a recent Mount Hood Diabetes Challenge, 10 of 12 health economic diabetes simulation models used UKPDS-OM risk equations [10].

Most diabetes simulation models comprise two main components: (1) event risk equations predicting death and diabetes-related complications, such as myocardial infarction and stroke, conditional on risk factor values and history of complications and (2) trajectories of risk factors [e.g. glycated haemoglobin (HbA_1c_), blood pressure] over the simulation period. Risk factor time path equations have been developed [11–13] that allow risk factor trajectories to be projected over a lifetime, which enables treatment effects on risk factors to be translated into differences in mortality and complications [3, 12]. By contrast, assuming constant risk factors or a constant annual increment or decrement throughout the whole simulation period is likely to underestimate complication rates [11].

Time path equations have been estimated for 13 clinical risk factors using data from the UKPDS trial collected between 1977 and 2002 [14]. While such equations provide useful historical information, there have been important changes in both the types of therapies available for treating type 2 diabetes [15] and patterns of clinical prescribing [16, 17]. Therefore, there is a need to re-estimate risk factor time path equations using more contemporary data that can be used to evaluate new therapies and technologies and inform decisions about regulatory approval and health technology assessment. These time paths are intended to extrapolate risk factors after the initial impact of the treatments being compared in the analysis; the time paths may include the effect of concomitant medications.

We used patient-level information from two large contemporary multinational randomised trials to develop new risk factor time path equations compatible with the UKPDS-OM2 and other diabetes models. Our study comprises four discrete steps: (1) estimating new time path equations to predict risk factors over time, (2) internally validating risk factor time path equations, (3) comparing the predictions of new models with the published equations [11] to quantify the QALY gains from improvements in risk factor control that have occurred over the last two decades and (4) updating diabetes model reference case simulations [2] to assess how incorporating these new risk-factor path equations will affect predicted outcomes for a variety of hypothetical interventions.

## Methods

### EXSCEL and TECOS Data

We pooled data from two randomised, placebo-controlled clinical cardiovascular outcome trials in people with type 2 diabetes: (1) the Exenatide Study of Cardiovascular Event Lowering [18, 19] (EXSCEL ClinicalTrials.gov NCT01144338), with 14,752 participants conducted between 2010 and 2017, and (2) the Trial Evaluating Cardiovascular Outcomes With Sitagliptin [20] (TECOS ClinicalTrials.gov, NCT00790205), with 14,671 participants conducted between 2009 and 2014. Both trials were pragmatic and allowed any concomitant medications (other than the drug class under investigation) to be used at the discretion of the usual care physician (see Supplementary Material Tables A1–A2 for a comparison between these trials).

Data from both arms of the two trials were combined to maximise generalisability and use all available data. Risk factor measurements performed < 6 months after starting randomised treatment were excluded from the analysis to exclude the initial effect of treatment. Within analyses for each risk factor, we excluded: (1) participants who did not provide any information on that risk factor at randomisation, (2) those who had information on that risk factor at the randomisation visit but not at follow-up visits, (3) those who withdrew from the study on the day of their randomisation visit and (4) and those who had missing data on ethnicity.

### Risk Factors

Our study focused on 11 risk factors: high-density lipoprotein cholesterol (HDL-C), low-density lipoprotein cholesterol (LDL-C), systolic blood pressure (SBP), HbA_1c_, heart rate, haemoglobin, body mass index (BMI), estimated glomerular filter rate (eGFR) and whether the patient had been diagnosed with peripheral vascular disease (PVD), atrial fibrillation (AF) or micro- or macroalbuminuria (ALB). Neither trial measured white blood cell count or post-randomisation smoking status, which are included in the UKPDS-OM2 [3].

Risk factors were analysed on a yearly basis, taking the average across the measurements in that year was used. Values outside predefined ranges [21] were omitted from the analysis.

#### Equations for Continuous Risk Factors

We applied linear dynamic model to estimate the time paths for HDL-C, LDL-C, HbA_1c_, haemoglobin, heart rate, SBP and BMI. Linear dynamic model in this study refers to the inclusion of the value of the risk factors in the previous period to capture the dynamic feature that previous values of risk factors affect current ones [11]. We used this model with random effects because it gave good predictions of risk factor values [within 95% confidence interval (CI) of observed values].^1^

The risk factor value for individual *i* in year *t* (${y}_{it}$) was:

1 $${y}_{it}={\phi }_{0}+{{\phi }_{1}y}_{it-1}+{\phi }_{2}{\text{y}}_{\text{i},0}+{\phi }_{3}{\text{sex}}_{i}+{{\phi }_{4}\text{ethnic}}_{ij}+{\phi }_{5}{\text{age}}_{i}+{\phi }_{6}\text{ln}\left({\text{duration of diabetes}}_{it}\right)+{\mu }_{i}+{\epsilon }_{it},$$

where ${y}_{i,t-1}$ was the previous year’s risk factor value, ${y}_{\text{i},0}$ was the first post-randomisation risk factor value and captured effect of the initial risk factors values on the subsequent time-path [11]; ${\text{ethnic}}_{ij}$ was a series of dummy variables, with ‘1’ indicating white, Black or Asian (oriental, Indian or other) and a baseline category of ‘other’ (Hispanic, Australian Aboriginal, Maori, Native Hawaiian, Pacific Islander, American Indian or Alaska Native), and age was age at randomisation. The natural log of ${\text{duration of diabetes}}_{it}$ was used as this was previously found to improve model fit [11]. The model included random effects by patient (${\mu }_{i}$), reflecting unobserved time-invariant characteristics, and an error component (${\epsilon }_{it}$).

#### Risk-Factor Equations for PVD, AF, ALB and eGFR

We applied multivariable parametric proportional hazard survival models to estimate the risk of developing PVD, AF, ALB and progressing to eGFR < 60 ml/min/1.73 m^2^. The underlying assumption is that once an individual progresses to one of these health states they can never leave. Several of the equations predicting diabetic events in UKPDS-OM2 incorporate eGFR as a spline variable with a knot at 60 ml/min/1.73 m^2^. Hence, to ensure robust predictions, the eGFR predictions must accurately represent not only the time paths of eGFR values but also the proportion of the population below the knot value (60 ml/min/1.73 m^2^).

We therefore modelled eGFR time paths with a two-part model, predicting whether eGFR was < 60 ml/min/1.73 m^2^ this year and then the exact eGFR value, conditional on covariates that included last year’s values. Monte Carlo simulation was used to convert probability predictions for individual patients into binary events (Supplementary Material 2). To predict a continuous eGFR value conditional on the eGFR < 60 ml/min/1.73 m^2^ prediction, we used two additional multivariable random effects Tobit autoregressive models of order one for eGFR values above or below 60. In the two separate Tobit models, we included the same covariates as the other continuous risk factors, and an upper limit of 60 ml/min/1.73 m^2^, a lower limit of 0 for eGFR <60 ml/min/1.73 m^2^ and a lower limit of 60 for eGFR ≥ 60 ml/min/1.73 m^2^. This two-step approach to predicting eGFR values has previously been shown to give the most accurate predictions of eGFR and events within UKPDS-OM2 [11].

For PVD, AF, ALB and the binary variable indicating eGFR < 60 ml/min/1.73 m^2^ (and ‘0’ otherwise), the parametric form (Weibull, exponential and Gompertz) was examined graphically and model choice was based on AIC. For all four outcomes, we selected a Weibull distribution. The proportional hazards assumption was tested by plotting Schoenfeld residuals and Cox–Snell semi-log graphs [22].

### Selection of Predictors

For all risk factor equations, we selected predictors based on two criteria: (1) as suggested by previous evidence [23], parsimonious models with fewer predictors were preferred over models with more predictors and (2) a good agreement between observed and predicted risk factor values (predicted values should be within 95% CI of observed values).

For the continuous risk equations, we included all covariates shown in Eq. 1 regardless of their statistical significance if they were shown to improve agreement between observed and predicted risk factor values. This ensured a parsimonious model informed by the covariates most likely to be available to other users.

For eGFR and the equations estimating time to PVD, AF and ALB, we followed the same approach as in the UKPDS-OM2 and Leal et al. [11]. The set of candidate covariates was initially informed by literature and expert opinion and included time-invariant factors (sex, age at diagnosis, ethnicity and smoking at baseline) and time varying clinical risk factors (SBP, HbA1c, BMI, HDL, heart rate and LDL). The multivariable models were initially fitted with all covariates; backwards stepwise regression at *P* < 0.05 was then used to select the significant covariates in the final models.

We explicitly excluded trial treatment allocation as a covariate in all risk equation models because the time path equations are intended to be applied to risk factor values from any diabetes dataset and any treatment. In sensitivity analysis, we re-estimated separate equations for each trial (TECOS and EXSCEL) and evaluated the impact of treatment allocation.

#### Mapping Out Risk Factor Trajectories

For all risk factors, predicted and observed time paths were plotted for the combined datasets to assess prediction accuracy and internal validity. We considered time paths to have good prediction accuracy if the predicted values lay within the 95% CI of observed values. Duration of diabetes was used as the time scale rather than time from randomisation, which allowed combining data on patients with different durations of diabetes together (similar to period life tables). For PVD, AF and ALB, we assessed calibration by plotting the observed cumulative incidence using the Kaplan–Meier estimator and comparing it with the predicted risk [24].

### QALY Gains Using Current and Previous Risk Equations

To estimate the health gains from improvements in risk factor management, pre-randomisation data for the placebo arms from both trials were extrapolated to 70 years using UKPDS-OM2. This analysis only included patients with non-missing data for all risk factors at both baseline and year 1 (*n* = 2579). Using coefficients estimated from Eq. (1), we first calculated values of risk factors in year 1 from pre-randomisation values and then simulated values from year 2 onwards. The same data were extrapolated using the time paths estimated by Leal et al. [11] and the difference in QALYs between the two simulations was estimated.

A bespoke version of UKPDS-OM2 was used which enabled the coefficients for binary risk factors to be modified. Baseline white blood cell count was imputed using a published algorithm [21]. Baseline data on smoking and white blood cell counts were extrapolated using the equations estimated by Leal et al. [11] in both scenarios, since these were not re-estimated in the current study. Default values for utilities and other parameters were used within these analyses and we ran 100,000 loops and no bootstraps (see Supplementary Material 3 for further details).

### Reference Simulation

Following recommendations of the Mount Hood Diabetes Challenge Network, we replicated the reference case simulations for the UKPDS-OM2 that were registered on the Mount Hood website (https://www.mthooddiabeteschallenge.com/registry) [25]. The registry includes a set of reference simulations that are intended to enable comparisons of models and increase model transparency [2].

The registry simulations were replicated using last observation carried forward (LOCF), trajectories estimated by Leal et al. [11] and those from Tables 1−2. Supplementary Material 4 gives further details.

Except where otherwise stated, all analyses were conducted using Stata version 17 (StataCorp, College Station, TX).

## Results

### Sample

In total, 28,608 participants (14,551 from EXSCEL and 14,057 from TECOS) were eligible for analysis. The average participant was 64 years old at randomisation and had lived with diabetes for around 16 years. The number of observations (person-years) that were available for analysis ranged from 41,928 (haemoglobin) to 94,068 (AF; Supplementary Material Tables A2–A3).

### Estimation of Risk Factor Time Path Equations

For continuous risk factors, both 1 year lagged risk factors and first recorded post-randomisation risk factor values had significant and positive effects on current values of risk factors in all risk equations (*P* < 0.01; Table 1). Older participants had significantly higher values of HDL-C and SBP, and lower values of LDL-C, HbA_1c_, haemoglobin, heart rate and BMI. Women had significantly higher values for all risk factors except haemoglobin, holding all else constant. Patients with higher first recorded values and lagged values of eGFR were less likely to develop eGFR < 60 ml/min/1.73 m^2^ (Table 2). SBP and duration of diabetes were significant and negatively correlated with eGFR in Tobit models.

**Table 1:** Coefficients for linear dynamic models with autoregression first order predicting annual risk factor values of continuous variables

	HDL-C	LDL-C	SBP	HbA1c	Haemoglobin	Heart rate	BMI
Value of *Y* in previous year	0.238***	0.295***	0.271***	0.456***	0.329***	0.283***	0.684***
	(0.018)	(0.011)	(0.006)	(0.009)	(0.016)	(0.007)	(0.023)
First recorded post-randomisation value of *Y* (> 6 months after treatment start)	0.506***	0.383***	0.291***	0.243***	0.462***	0.346***	0.289***
	(0.021)	(0.012)	(0.007)	(0.009)	(0.017)	(0.007)	(0.023)
ln(duration of diabetes)	− 0.001	− 0.037***	0.034	0.083***	− 0.096***	− 0.186**	0.000
	(0.002)	(0.008)	(0.122)	(0.009)	(0.015)	(0.075)	(0.013)
Age at randomisation	0.001***	− 0.004***	0.027***	− 0.012***	− 0.010***	− 0.059***	− 0.008***
	(0.000)	(0.001)	(0.008)	(0.001)	(0.001)	(0.005)	(0.001)
Female	0.055***	0.100***	0.356***	0.031***	− 0.177***	0.560***	0.039**
	(0.003)	(0.009)	(0.136)	(0.010)	(0.018)	(0.085)	(0.015)
Ethnicity (reference group: other^§^)							
White ethnicity	0.019***	− 0.001	0.365	− 0.082***	− 0.037	0.316**	0.022
	(0.005)	(0.019)	(0.271)	(0.021)	(0.037)	(0.155)	(0.028)
Black ethnicity	0.045***	0.032	0.780*	0.065*	− 0.208***	0.376	-0.079*
	(0.008)	(0.027)	(0.442)	(0.035)	(0.049)	(0.261)	(0.044)
Asian (oriental and other) ethnicity	0.015**	− 0.037*	− 0.416	− 0.091***	− 0.081*	1.389***	− 0.078***
	(0.006)	(0.020)	(0.307)	(0.023)	(0.042)	(0.184)	(0.030)
Constant	0.188***	1.027***	56.461***	2.945***	3.698***	30.755***	1.219***
	(0.014)	(0.044)	(0.850)	(0.064)	(0.149)	(0.563)	(0.094)
*R*^2^	0.60	0.51	0.30	0.50	0.65	0.40	0.95
Observations	37,792	35,554	54,790	51,453	22,657	53,928	53,885
Number of individuals	18,499	17,523	24,115	22,741	12,116	23,867	23,747

Supplemental Material 2 describes how to use these coefficients to predict risk factors for individual patients, with examples

^§^Reference group for ethnicity is Hispanic, Aboriginal (Australia), Maori (New Zealand), Native Hawaiian or Other Pacific Islander, Indian (American) or Alaska Native. Coefficient values with a larger number of decimal places and using white ethnicity as the reference group are available from the corresponding author on request.

Robust standard errors in parentheses; ***P < 0.01, **P < 0.05, *P < 0.1

BMI, body mass index; HbA_1c_, glycated haemoglobin; HDL-C, high-density lipoprotein cholesterol; LDL-C, low-density lipoprotein cholesterol; SBP, systolic blood pressure

**Table 2:** Sample size, functional form, parameters and beta coefficients (standard error) for equations estimating probability of binary variables and eGFR values

Variables	AF	ALB	PVD	eGFR < 60 (binary)	eGFR < 60 (continuous)	eGFR ≥ 60 (continuous)
Function form	Weibull	Weibull	Weibull	Weibull	Tobit model	Tobit model
Age at randomisation	0.060***	0.011**	0.030***		− 0.100***	− 0.230***
	(0.006)	(0.005)	(0.008)		(0.016)	(0.011)
Female	− 0.576***	− 0.298***				
	(0.124)	(0.092)				
White ethnicity	1.073***	0.291***	1.120***			
	(0.198)	(0.104)	(0.216)			
Black ethnicity	1.150***					
	(0.303)					
Smoking at randomisation			0.465***			
			(0.124)			
BMI last year	0.058***	0.028***				
	(0.007)	(0.006)				
First recorded value of eGFR			− 0.037***	0.232***	0.446***	
				(0.002)	(0.015)	(0.010)
eGFR last year				− 0.032***	0.459***	0.303***
				(0.003)	(0.016)	(0.011)
SBP last year		0.011***			− 0.025***	− 0.011**
		(0.003)			(0.007)	(0.005)
Lag HDL last year	− 0.487***					
	(0.176)					
HbA_1c_ last year		0.102***	0.151***			
		(0.034)	(0.054)			
Ln(duration of diabetes)					− 1.335***	− 0.753***
					(0.228)	(0.164)
Constant	− 11.269***	− 8.590***	− 11.596***	0.706**	36.058***	36.317***
	(0.572)	(0.614)	(0.803)	(0.299)	(1.611)	(1.108)
ln(*Γ*)	0.191**	0.204***	0.443***	0.078		
	(0.076)	(0.060)	(0.090)	(0.060)		
Sigma: Standard error of the forecast (estimated using predict stdf, stdf)					11.888	13.839
Observations	46,281	34,627	52,412	53,765	37,997	37,997
Number of events	416	596	219	755	N/A	N/A

Coefficient values with a larger number of decimal places and using white ethnicity as the reference group are available from the corresponding author on request. Supplemental Material 2 describes how to use these coefficients to predict risk factors for individual patients, with examples.

Robust standard errors in parentheses; ***P < 0.01, **P < 0.05, *P < 0.1

AF, whether the patient has been diagnosed with atrial fibrillation; ALB, whether the patient has been diagnosed with micro- or macro-albuminuria; BMI, body mass index; eGFR, estimated glomerular filter rate; HbA_1c_, glycated haemoglobin; HDL-C, high-density lipoprotein cholesterol; PVD, peripheral vascular disease; SBP, systolic blood pressure. The baseline category for ethnicity is any non-white or non-Black ethnicity

Older patients were more likely to be diagnosed with AF, ALB and PVD, while women were less likely to be diagnosed with AF and ALB (Table 2). The probability of being diagnosed with AF was significantly affected by smoking, black or white ethnicity and 1 year lagged values of BMI. The probability of being diagnosed with ALB was significantly affected by white ethnicity, lagged values of BMI, SBP and HbA_1c_. The probability of being diagnosed with PVD was significantly affected by smoking at baseline and HbA_1c_. For users interested in trial-specific risk factor trajectories, we report separate equations for each trial in the supplementary materials (Tables A5–A6). Finally, Supplemental Material 2 describes how the equations are to be used to predict risk factor progression.

### Internal and Cross Validation of Risk Factor Time Path Equations

**Fig. 1:**
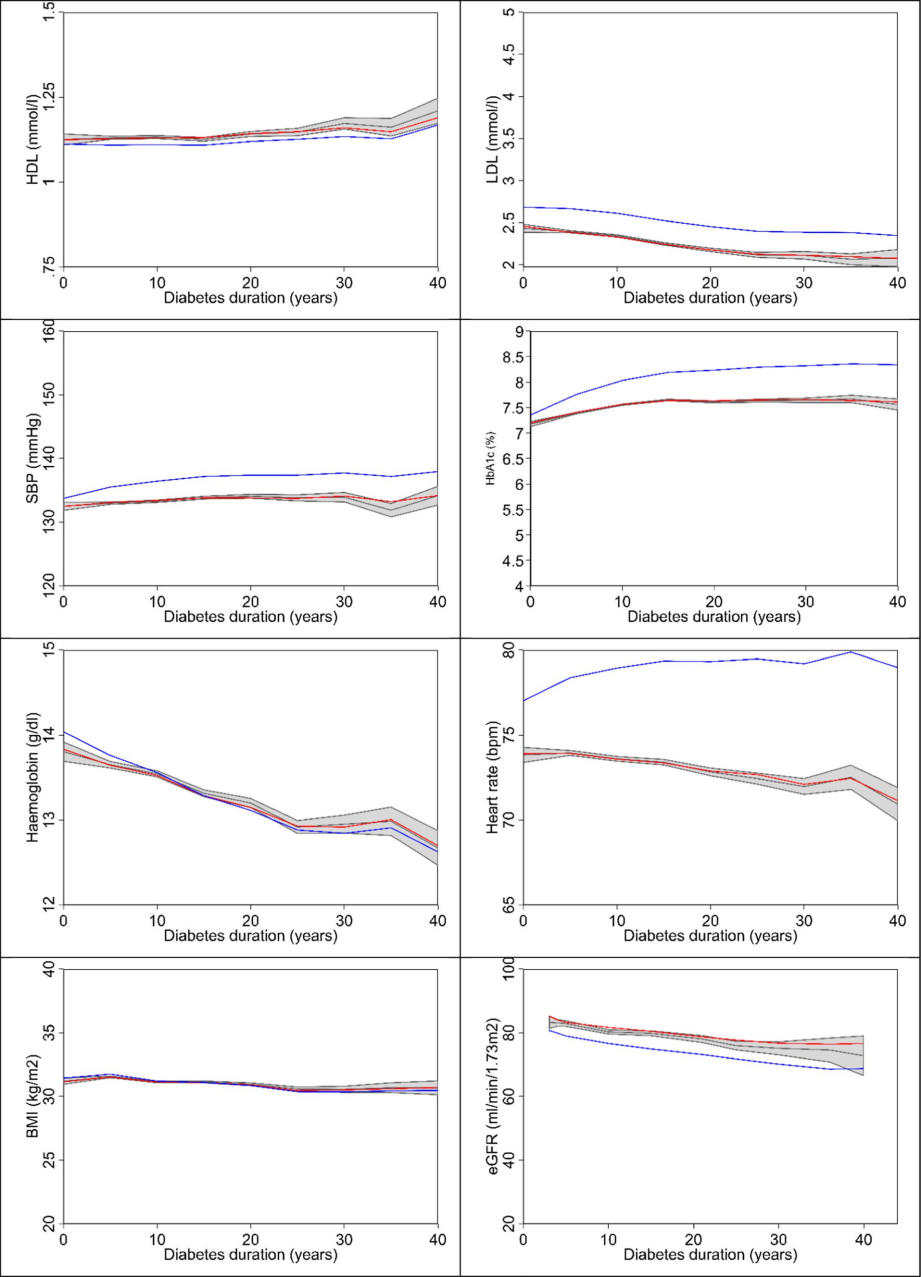
Observed values with 95% confidence intervals (grey area) and simulated time paths for continuous risk factors estimated in the current study (red line) and by Leal et al. [11] (blue line). Each patient was followed for up to 6 years, and patients are combined to plot risk factors by diabetes duration. BMI, body mass index; eGFR, estimated glomerular filtration rate; HbA_1c_, glycated haemoglobin; HDL-C, high-density lipoprotein cholesterol; LDL-C, low-density lipoprotein cholesterol; SBP, systolic blood pressure

The predicted values of continuous risk factors were within the 95% CIs of the observed values for each risk factor (Fig. 1), suggesting a good agreement between observed and modelled risk factors. HDL-C and HbA_1c_ increased slightly with duration of diabetes whilst SBP and BMI were relatively stable. LDL-C, haemoglobin and heart rate showed downward trends.

The coefficients estimated on the pooled dataset (Table 1) perform well for both TECOS and EXSCEL for all risk factors, except HbA_1c_ (Supplementary Materials Fig. A1). Time paths of predicted and observed risk factor values were further compared by quintiles based on the first observed risk factors value (Supplementary Materials Fig. A2 and Supplementary Materials Table A4). In general, predicted risk factor values were within 95% CIs of observed values in each quintile.

**Fig. 2:**
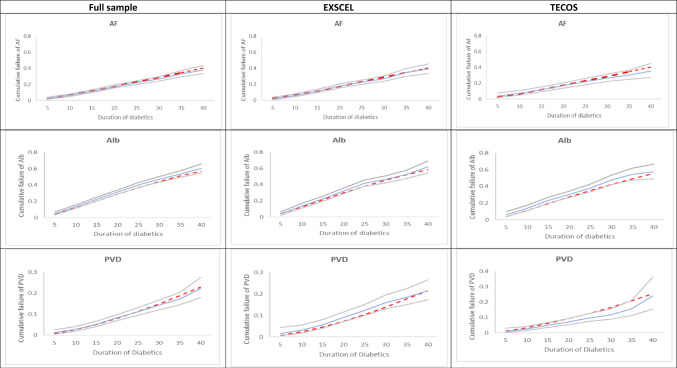
Kaplan–Meier estimates of observed (blue line) and simulated* (red line) cumulative failure of AF, ALB and PVD. Observed 95% confidence intervals (Cis) are represented using grey lines. Each patient was followed for up to 6 years, and patients were combined to plot risk factors by diabetes duration. Cumulative incidence is 1 minus Kaplan–Meier. AF, whether the patient has been diagnosed with atrial fibrillation; ALB, whether the patient has been diagnosed with micro- or macro-albuminuria; EXSCEL, Exenatide Study of Cardiovascular Event Lowering; PVD, peripheral vascular disease; TECOS, Trial Evaluating Cardiovascular Outcomes With Sitagliptin

Predicted time paths of risk factors using coefficients from Leal et al. [11] were compared graphically with those from the current study (Fig. 1). Except for BMI and haemoglobin, time paths using previous risk equations showed poor agreement with observed risk factor time paths. The predicted cumulative incidence of AF, ALB and PVD was consistent with observed events: both among the full sample, and the EXSCEL and TECOS samples separately (Fig. 2). Cross-validation suggested that for the models of LDL, HDL, SBP, haemoglobin, heart rate and BMI predictions based on coefficients estimated on the EXSCEL trial lie within the 95% CI of observed outcomes for TECOS patients (vice versa; Supplementary Materials Fig. A4). However, for HbA1c, values predicted based on the other study lay outside the 95% CI, with EXSCEL coefficients predicting HbA1c values to be 0.1% higher than those from TECOS. Sensitivity analyses suggested that exenatide may affect SBP and heart rate trajectories while sitagliptin may affect HbA1c trajectories (Supplementary Materials 5).

### QALY Gains Using Updated Risk Equations and Previous Ones

**Fig. 3:**
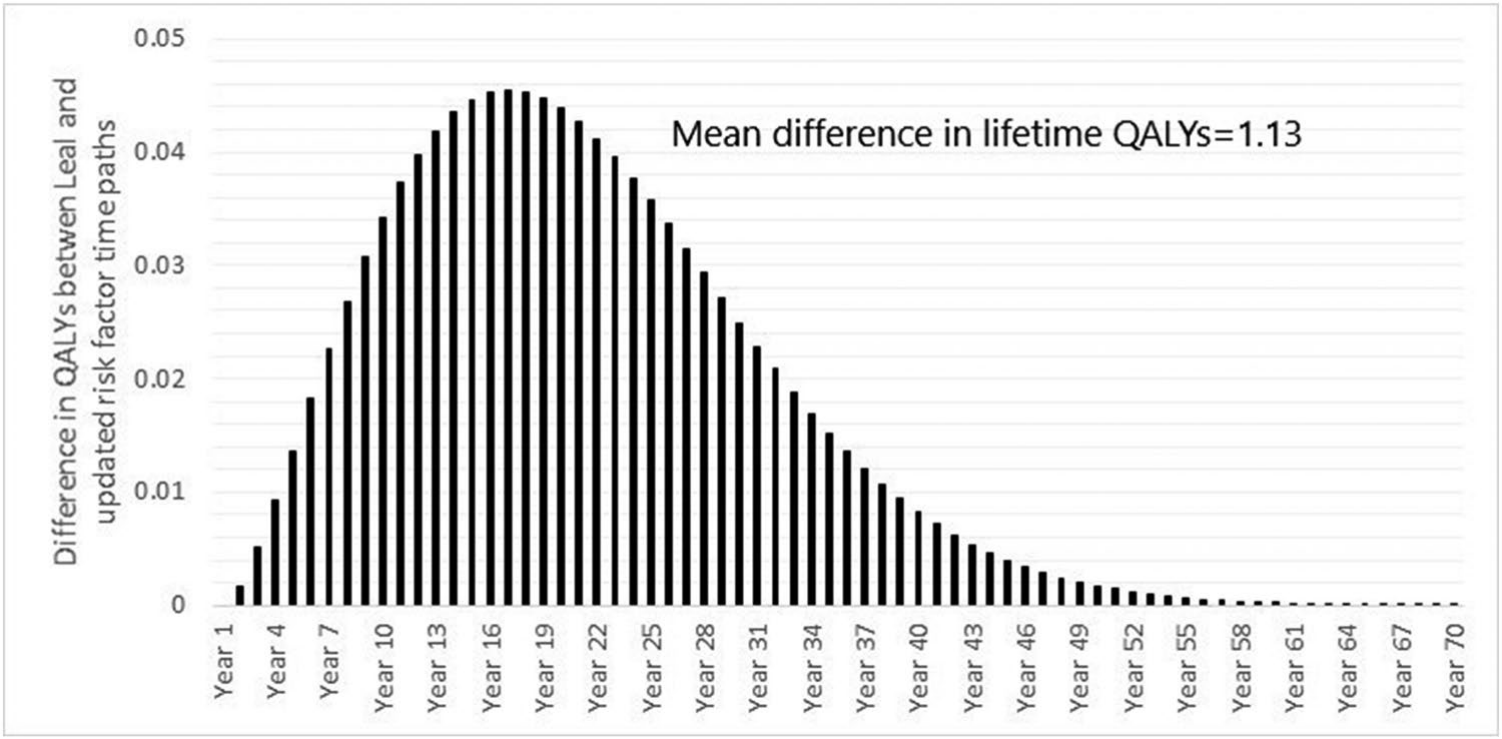
Mean QALY gains per year using current and previous risk equations for the 2563 patients randomised to placebo who had complete data on all risk factors at randomisation and in the first-year post-randomisation. The graph plots the difference in QALYs using updated risk equations and previous ones by Leal et al. [11] at each time point. QALY, quality-adjusted life-year.

UKPDS-OM2 was used to simulate QALYs over 70 years using both the risk factor trajectories equations in the current study and those of Leal et al. [11]. On average, the cohort was predicted to accrue 9.84 (standard deviation 4.64) QALYs using risk factor equations estimated on the UKPDS sample [11], compared with 10.98 (standard deviation 5.14) QALYs using the risk factor equations estimated in the current paper (Fig. 3). This equates to a gain of 1.13 (95% CI 0.90–1.36) QALYs per patient (12%; *P* < 0.001 in paired *t* test).

### Mount Hood Reference Case Simulations

**Fig. 4:**
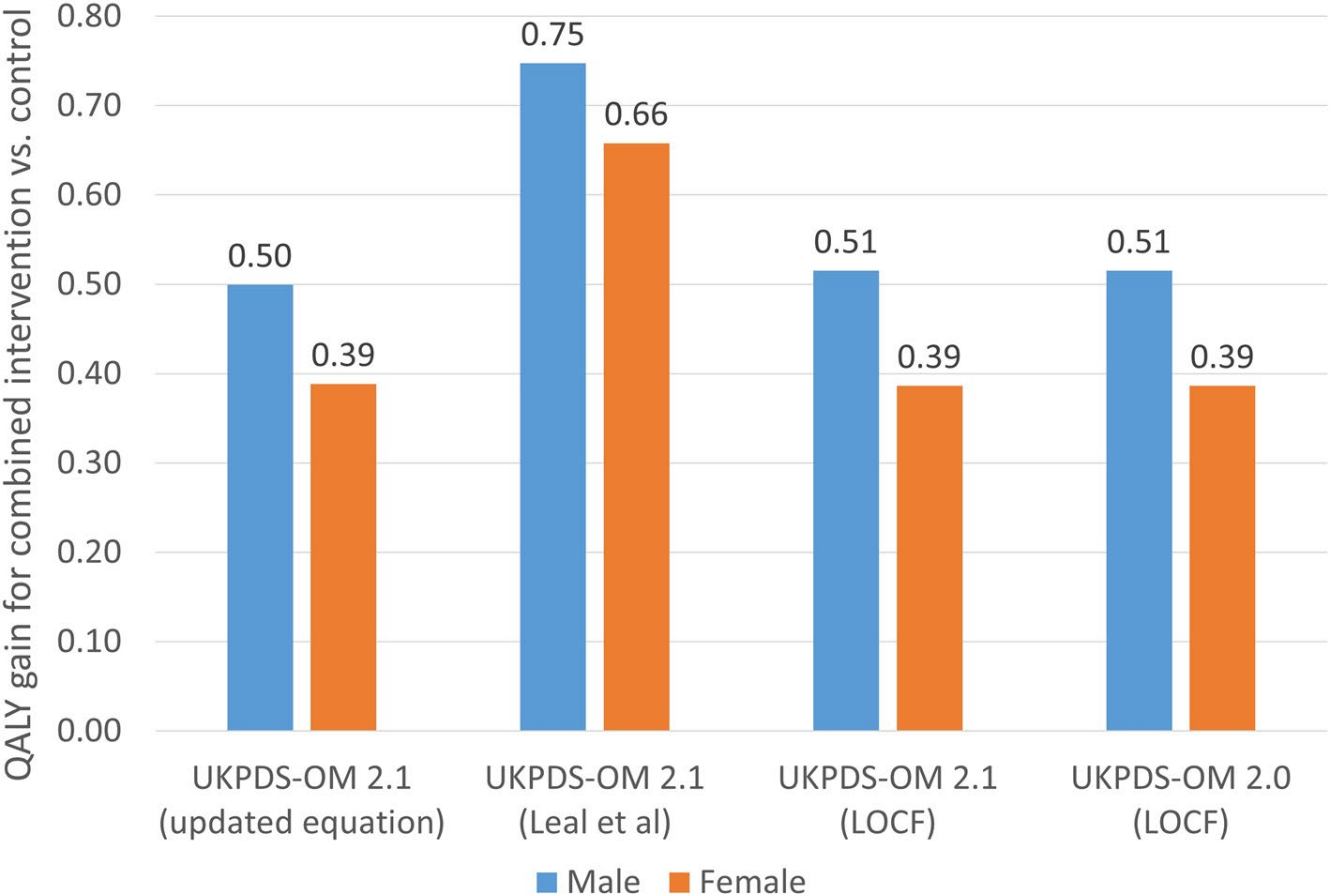
Results of the Mount Hood reference case simulation for: UKPDS-OM version 2.0 assuming: last observation carried forward (LOCF), UKPDS-OM version 2.2 using updated risk factor trajectories estimated in the current paper, UKPDS-OM version 2.2 using risk factor trajectories estimated by Leal et al. [11] and UKPDS-OM version 2.2 assuming LOCF. This shows the difference in QALYs between the hypothetical ‘combined’ intervention (simultaneously reducing HbA_1c_ by 0.5%, systolic blood pressure by 10 mmHg, low-density lipoprotein by 0.5 mmol/l and body mass index by one unit) compared with control, for the Mount Hood registry reference patients [25]. HbA1c glycated haemoglobin; LOCF, last observation carried forward; QALY, quality-adjusted life-year; UKPDS-OM, United Kingdom Prospective Diabetes Study Outcomes Model

With LOCF, the model used in our simulation produced results for this reference simulation that were identical to those reported in the Mount Hood registry on 5 October 2018 [25] other than Monte Carlo error. Compared with LOCF, applying risk factor trajectories reduced the QALYs accrued by all reference patients and increased the QALYs gains from reducing HbA_1c_, blood pressure, LDL-C and BMI by the fixed increments specified for the reference case simulation [25] (Table A10, Fig. 4). The QALYs gains were smaller for the risk factor trajectories estimated in the current study than the trajectories estimated by Leal et al. [11]: reducing men’s HbA_1c_, blood pressure, LDL-C and BMI together gained 0.75 QALYs with trajectories from Leal et al. [11] and 0.50 QALYs with trajectories from the current study and 0.51 QALYs using LOCF.

## Discussion

We estimated a set of contemporary risk factor time path equations for type 2 diabetes. These equations were derived from two large clinical trials covering 48 countries with > 80,000 person-years of follow-up. We show the equations and predictions to be clinically plausible and internally valid within and between the trials. Furthermore, we show gains of 1.13 QALYs associated with improvements in risk factor trajectories relative to the UKDPS time period.

The equations have been estimated so that they can be integrated into the UKPDS-OM2 and other diabetes simulation models, to facilitate predictions of diabetes-related complications and death consistent with contemporary diabetes populations. Internal validation of the updated equations gave much better predictions than those estimated on historical UKPDS data [11]. In particular, the older equations predicted markedly higher HbA1c levels than the observed values or new equations. However, the older models continued to give reasonable predictions for BMI and haemoglobin, which may suggest that these risk factor time paths have improved less over time than for other risk factors. Earlier studies omitted important risk factors (e.g. eGFR) and more recent trends in metabolic control [13]. With our multinational data, we also estimated coefficients for more ethnic groups than previous studies [11]. Simple risk factor equations mean that the covariates within our models are likely to be available in other datasets.

Although the equations are relatively parsimonious, they showed good fit within the two trials. Between-trial cross-validity was good for LDL, HDL, SBP, haemoglobin, heart rate and BMI, suggesting that the coefficients estimated on the pooled dataset are most informative. For HbA_1c_, the observed trajectory differed between EXSCEL and TECOS and cross-validity was not as good. This is likely the result of differences between trial protocols: the TECOS trial was designed to optimise the likelihood of achieving glycaemic equipoise [26], whereas in EXSCEL, this was not a requirement [19]. Hence, we also provide trial-specific equations for those interested in replicating the trajectories observed in a given trial or a clinical setting.

These equations can inform economic evaluations of diabetes management strategies, which will improve risk stratification for guiding healthcare resource allocation and targeting treatment approaches. Current health economic diabetes simulation models typically capture treatment effects via changes in ≥ 1 risk factor. For example, the effect of glucose-lowering drugs is usually simulated through changes in HbA_1c_ relative to the trajectory observed in usual care. To remove any changes in risk factor values related to study interventions or study participation, we excluded data on the first 6 months of the trial when estimating our models and focused on estimating subsequent trajectories. We also controlled for lagged values of risk factors and demographic factors such as sex, age and ethnicity. Decision modellers are encouraged to use their own data on treatment effect in the first year and then use our estimated models afterwards (Supplementary Material 2). Our risk factor time path equations are intended to simulate background time-paths that could be applied to patients on any stable treatment. The time paths reflect the natural history of risk factors and contemporary patterns of disease management and concomitant medication. Most risk factors were not affected by treatment allocation; treatment-specific models are presented for those that may be sensitive (Supplementary Materials, Table A11).

Although models, such as UKPDS-OM2, have performed reasonably well on external validation, they appear to overestimate mortality and myocardial infarction rates [21]. Updating risk factor time paths may go some way to improving the prediction accuracy of diabetes simulation models, further research may also be needed to update event equations.

The reference case simulations highlight the impact of time path equations on decision-making in diabetes. Our contemporary time-paths increased incremental QALYs by 10–20% compared with the previous reference simulation, which assumed LOCF but decreased incremental QALYs compared with historic risk factor trajectories [11]. These QALY gains exclude the impact of changes in smoking cessation rates. Although we estimated these QALY gains using a common patient sample and used similar methods to Leal et al. [11], it is possible that part of the difference in trajectories could be attributable to differences in methodology or inclusion criteria. The QALY gains reinforce the importance of using contemporary risk factor time path equations in economic evaluation that are relevant to the target diabetes population. Furthermore, as the standards of diabetes care are likely to continue to improve, there is a strong case for updating the equations periodically to reflect changes in clinical practice. While we found that region dummies worsened prediction accuracy, future research should further explore variation in these time-path equations in different settings, populations and regions. Estimating future equations using a similar approach to that adopted here would facilitate comparison across different estimates and allow easy integration into existing diabetes simulation models that are based on the UKPDS-OM2 structure.

Our analysis has a number of limitations. First, the trials used provided only 6 years’ follow-up; however, the wide variation in diabetes duration at baseline allowed us to estimate risk factor trajectories over 40 years after diagnosis. Second, the trajectories described by these equations represent the outcome of a mixture of treatments and natural history over time in EXSCEL and TECOS. Analysing the impact of specific treatments on risk factors was beyond the scope of the current study, especially for newer agents such as sodium-glucose cotransporter-2 inhibitors and glucagon-like peptide-1 receptor agonists that deliver cardiovascular and renal risk reductions by mechanisms other than improving conventional risk factor values [27, 28]. Further research is needed to assess whether risk factor trajectories beyond the first year of treatment are different in populations receiving the newer drugs: especially for BMI and HbA1c. Third, the observed trajectory of some risk factors may not be representative of a typical diabetes patient given the potentially more intense management in the trials. However, both EXCSEL and TECOS both used highly pragmatic designs with few restrictions on concomitant medications, little additional monitoring over usual care and wide-ranging eligibility criteria [19, 26]. There is a shortage of long-term studies collecting regular data on all risk factors and the early years of follow-up in long studies are unavoidably historical. Fourth, all TECOS participants [26] and 73% of EXSCEL participants [29] had cardiovascular disease at baseline; in principle this could affect risk factor trajectories, although we are not aware of any evidence for this. Further research externally validating our equations in other contemporary cohorts is recommended, following the Mount Hood tradition of extensive external validation [30]. Finally, white blood cell counts and post-baseline data on smoking status were not collected in EXSCEL or TECOS. As a result, UKPDS-OM2 users will continue to rely on older equations for these two risk factors based on the UKPDS data [11].

## Conclusions

Our new equations give modellers and the wider research community a useful additional tool to simulate the long-term effects of type 2 diabetes and its therapies. The parsimonious estimation approach encourages their replication across datasets and populations facilitating the sharing and comparison of knowledge across researchers.

### Supplementary Information

Below is the link to the electronic supplementary material.

Supplementary file1 (PDF 2170 KB)

## Footnotes

1 We did not use linear dynamic model with fixed effects because they are not able to estimate time-invariant variables such as gender and ethnicity. We did not use system generalised method of moments (GMM) models because they are sensitive to the correct specification of temporal lags (how many lagged values of risk factors (e.g. values of risk factors in previous one year or two years should be included). Particularly, even slight alterations in temporal lags lead to different coefficients and even changes in the significance of the coefficients. Given that prediction of risk factors is the primary objective of our current study, we opted for a parsimonious model over GMM model, to ensure the stability of coefficients.

## Declarations

### Data availability Statement

Requests for data access and proposals for analyses of EXSCEL and TECOS data can be submitted to the respective study Publications Committee using instructions found at https://www.dtu.ox.ac.uk/exscel/ and https://www.dtu.ox.ac.uk/TECOS/.
