# Supplementary material for: Estimating Risk Factor Time Paths Among People With Type 2 Diabetes And QALY Gains From Risk Factor Management
Source: Pharmacoeconomics. Author manuscript; Available in PMC 2024 Aug 25. (PMC11344020; doi:10.1007/s40273-024-01398-4)
Supplement: Supplementary Materials [file EMS196789-supplement-Supplementary_Materials.pdf]

Electronic Supplementary Material

Estimating Risk Factor Time Paths Among People With Type 2 Diabetes And QALY Gains From Risk Factor Management

Pharmacoeconomics 2024 https://doi.org/10.1007/s40273-024-01398-4

Ni Gao, MBBS, PhD,[1,2] Helen A. Dakin, DPhil,[1] Rury R. Holman, FRCP[3], Lee-Ling Lim, MBBS, PhD[4-6], José Leal, DPhil,[1*] Philip Clarke, PhD [1*]
* These authors contributed equally

[1] Health Economics Research Centre, University of Oxford, Oxford, UK
[2] Centre for Health Economics, University of York, York, UK
[3] Diabetes Trials Unit, Radcliffe Department of Medicine, University of Oxford, Oxford, UK
[4] Department of Medicine, Faculty of Medicine University of Malaya, Kuala Lumpur, Malaysia
[5] Department of Medicine and Therapeutics, The Chinese University of Hong Kong, Hong Kong SAR
[6] Asia Diabetes Foundation, Hong Kong, SAR

Corresponding author: Helen Dakin, helen.dakin@dph.ox.ac.uk

	Page
Supplementary material 1: Additional results	2
Supplementary material 2: Instructions on how to use the coefficients to predict risk factors	15
Supplementary material 3: Methods for QALY gains using current and previous risk equations extrapolated using UKPDS-OM2	21
Supplementary material 4: Methods and results of reference simulation	22
Supplementary material 5: Evaluating impact of randomised treatment group on time paths ≥12 months after start of treatment	28
References for supplementary material	30

Supplementary material 1: Additional results

Table A1. Comparison of the trials used in the study

**Study Name**	**EXSCEL [1, 2]**	**TECOS [3]**
**Study Calendar Time**	2010-2017	2009-2014
**Target Population**	Type 2 diabetes with a glycated haemoglobin (HbA_{1c}) between ≥7.0% and ≤10.0% on stable dose of oral antihyperglycemic agents for at least 3 months. eGFR ≥30 mL/min/1.73 m^2 with approximately 70% having previous cardiovascular disease	Type 2 diabetes with HbA_{1c} between ≥6.5% and ≤8.0% on stable dose of metformin, pioglitazone, or a sulfonylurea (either monotherapy or dual combination therapy) for at least 3 months. eGFR ≥30 mL/min/1.73 m^2.
**Duration of diabetes**	13.1 (standard deviation 8.3) years	11.6 (standard deviation 8.1) years
**Number of participants**	14,752	14,671
**Median follow up**	3.2 (maximum 6.8) years	3.0 (maximum 5.7) years
**Method for estimating eGFR**	Modification of Diet in Renal Disease (MDRD) method	Modification of Diet in Renal Disease (MDRD) method

Abbreviations: eGFR, estimated glomerular filter rate; EXSCEL, Exenatide Study of Cardiovascular Event Lowering; TECOS, Trial Evaluating Cardiovascular Outcomes With Sitagliptin.

Table A2. Summary of risk factors averaged over all years. Patient characteristics for each trial at randomisation have been reported[4,5] previously [4, 5].

	EXSCEL mean (SD) / %(n)	TECOS mean (SD) / %(n)	Pooled sample mean (SD) / %(n)	Pooled sample Number of Individuals	Pooled sample Number of observations
HDL-C (mmol/l)	1.1 (0.3)	1.1 (0.3)	1.1 (0.3)	24420	64371
LDL-C (mmol/l)	2.4 (1.0)	2.3 (0.9)	2.3 (0.9)	23623	61387
SBP (mmHg)	133.5 (15.2)	133.5 (16.6)	133.5 (15.9)	27182	83422
HbA_{1c} (%)	7.8 (1.4)	7.3 (1.1)	7.5 (1.7)	26192	79248
Haemoglobin (g/dL)	13.6 (1.6)	13.1 (1.9)	13.4 (1.7)	17758	41928
Heart rate (bpm)	74.7 (9.9)	72.3 (10.7)	73.5 (10.4)	27085	82550
BMI (kg/m^2)	32.4 (6.3)	30.0 (5.6)	31.2 (6.1)	26905	82312
eGFR (ml/min/1.73m^2)	75.9 (23.5)	72.8 (21.4)	74.3 (22.5)	26957	78658
PVD	0.28%	0.33%	0.23%	23608	84633
AF	0.71%	0.51%	0.62%	26222	94068
ALB	1.20%	1.08%	1.14%	16691	59114

Abbreviations: AF, whether the patient has been diagnosed with atrial fibrillation; ALB, whether the patient has been diagnosed with micro- or macroalbuminuria; BMI, body mass index (BMI); eGFR, estimated glomerular filtration rate; EXSCEL, Exenatide Study of Cardiovascular Event Lowering; HbA_{1c}, glycated haemoglobin; HDL-C, high-density lipoprotein cholesterol; LDL-C, low-density lipoprotein cholesterol; PVD, peripheral vascular disease; SBP, systolic blood pressure; SD, standard deviation; TECOS, Trial Evaluating Cardiovascular Outcomes With Sitagliptin.

Table A3. Mean, SD and number of individuals (N) for each continuous risk factor during six follow-up years

		Follow-up years					
**Risk factors**		**1**	**2**	**3**	**4**	**5**	**6**
HDL (mmol/l)	N	19,546	19,846	13,971	7,914	2,667	427
	Mean	1.13	1.13	1.13	1.14	1.15	1.17
	SD	0.32	0.31	0.32	0.32	0.32	0.33
LDL (mmol/l)	N	18,556	18,900	13,323	7,578	2,610	420
	Mean	2.35	2.31	2.29	2.31	2.29	2.18
	SD	0.96	0.94	0.92	0.95	0.97	0.83
SBP (mmHg)	N	26,818	24,398	17,802	10,175	3,664	565
	Mean	133.55	133.88	133.59	133.23	131.97	129.29
	SD	15.92	16.03	15.89	15.75	15.73	14.97
HbA_{1c} (%)	N	24,933	23,360	17,082	9,873	3,474	526
	Mean	7.47	7.54	7.53	7.55	7.50	7.74
	SD	1.22	1.26	1.27	1.31	1.27	1.51
Haemoglobin (g/dL)	N	12,518	13,146	9,040	5,143	1,763	318
	Mean	13.51	13.41	13.35	13.35	13.35	13.36
	SD	1.71	1.76	1.76	1.73	1.69	1.80
Heart rate (bpm)	N	11,046	24,141	17,624	10,076	3,629	554
	Mean	74.89	73.40	73.29	73.37	73.52	74.80
	SD	11.15	10.42	10.48	10.35	10.29	10.49
BMI (kg/m^2)	N	26,473	24,023	17,567	10,060	3,635	554
	Mean	31.30	31.27	31.06	31.05	30.95	33.66
	SD	6.06	6.06	6.02	6.08	6.23	6.75
eGFR (ml/min/1.73m^2)	N	23,481	23,699	17,463	10,028	3,471	516
	Mean	75.72	74.31	73.71	72.72	72.49	74.32
	SD	22.60	22.22	22.54	22.69	22.96	24.08

Table A4. Mean and SD for each fifth of the population (first observed value post randomisation) of the continuous risk factors

Risk factors	Quintile	Mean	SD	N
HDL (mmol/l)	1	0.76	0.10	25281
	2	0.95	0.04	25562
	3	1.08	0.04	26160
	4	1.24	0.05	25768
	5	1.61	0.29	25737
LDL (mmol/l)	1	1.23	0.24	24497
	2	1.76	0.12	24820
	3	2.17	0.12	24482
	4	2.69	0.19	25214
	5	3.82	0.77	24754
SBP (mmHg)	1	110.90	6.79	23796
	2	124.06	3.10	33945
	3	132.35	2.18	31776
	4	140.18	2.06	27210
	5	155.56	10.37	32647
HbA_{1c} (%)	1	6.05	0.36	25644
	2	6.72	0.14	27240
	3	7.25	0.17	32678
	4	7.91	0.22	28341
	5	9.43	1.05	29024
Haemoglobin (g/dL)	1	10.87	1.25	17631
	2	12.70	0.28	17785
	3	13.56	0.23	18977
	4	14.39	0.26	18642
	5	15.70	0.74	18719
Heart rate (bpm)	1	60.12	4.35	29697
	2	68.37	1.41	27368
	3	73.06	1.36	29926
	4	78.37	1.72	31780
	5	88.53	6.59	29852
BMI (kg/m^2)	1	24.07	1.84	29544
	2	27.88	0.83	29547
	3	30.61	0.80	29545
	4	33.79	1.10	29547
	5	40.63	4.70	29547
eGFR ($ml/min/1.73m^2$)	1	56.07	9.17	14968
	2	70.17	2.74	14580
	3	79.61	2.85	15373
	4	90.16	3.35	14886
	5	111.61	14.03	15065

Figure A1. Continuous risk factor values for the EXSCEL and TECOS study populations separately, using the time path models that were estimated on the pooled dataset (coefficients in Table 1). Observed values (black line) are shown with 95% confidence intervals (grey area) and simulated (red line) time paths.

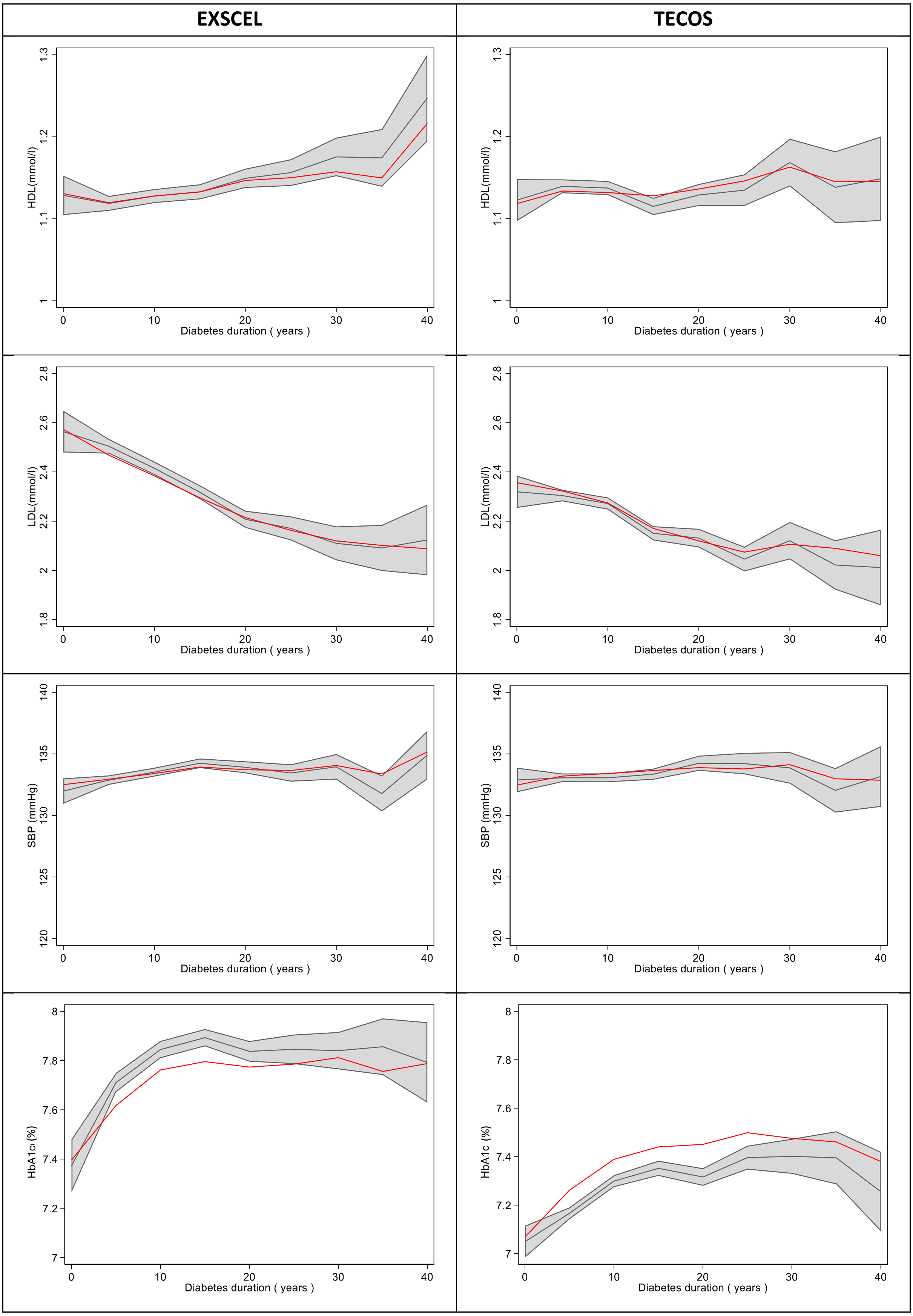

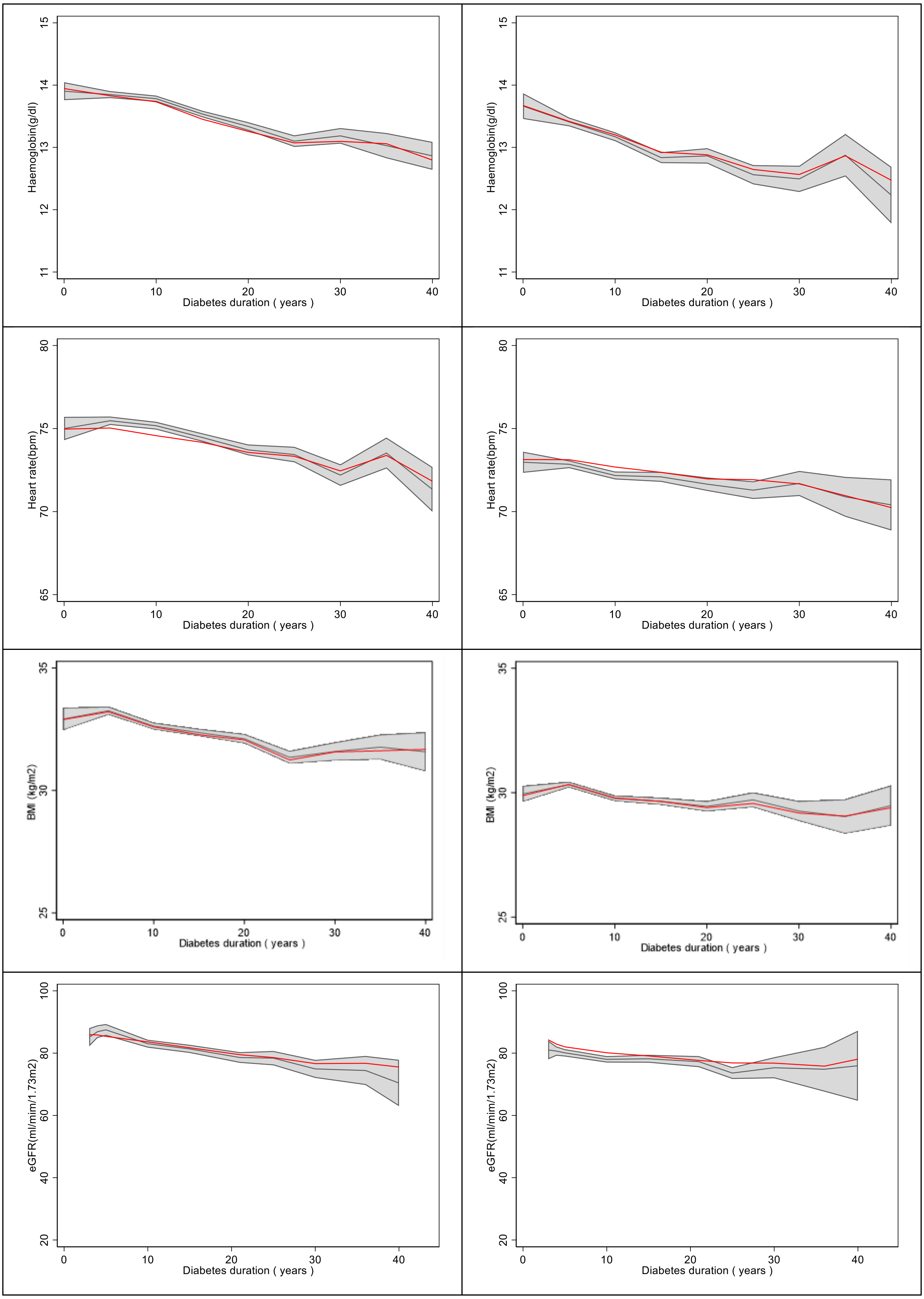
Haemoglobin(g/dl)
Diabetes duration (years)
Haemoglobin(g/dl)
Diabetes duration (years)
Heart rate(bpm)
Diabetes duration (years)
Heart rate(bpm)
Diabetes duration (years)
BMI (kg/m2)
Diabetes duration (years)
BMI (kg/m2)
Diabetes duration (years)
eGFR(ml/mim/1.73m2)
Diabetes duration (years)
eGFR(ml/mim/1.73m2)
Diabetes duration (years)

Figure A2. Observed values with 95% confidence intervals (grey area) and simulated* (red line) time paths by quintile of continuous risk factors

Q	HDL	LDL	SBP
1	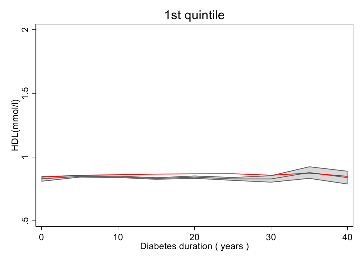	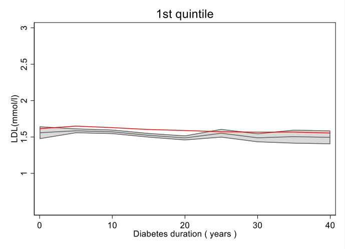	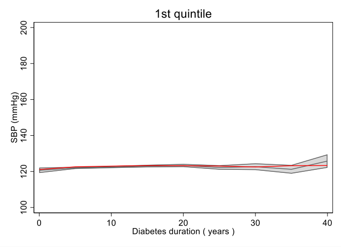
2	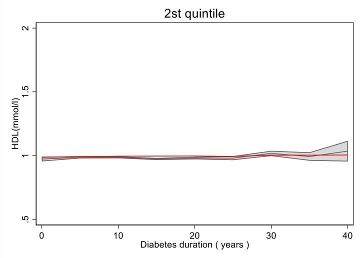	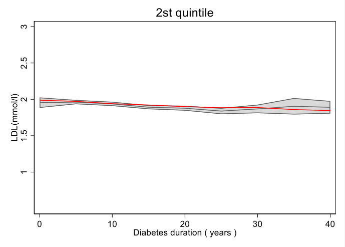	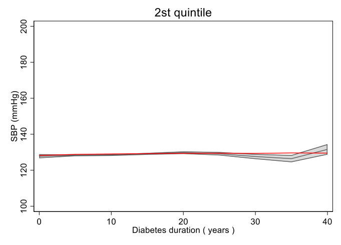
3	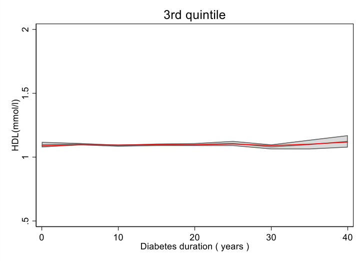	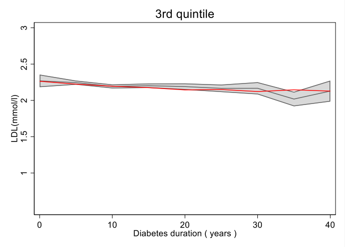	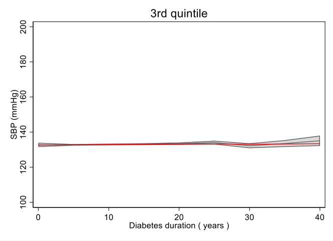
4	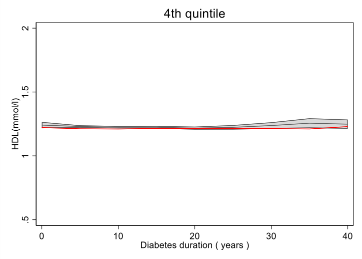	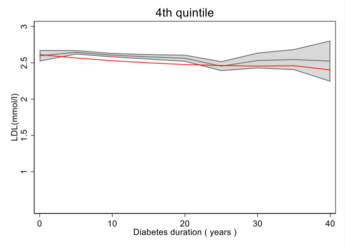	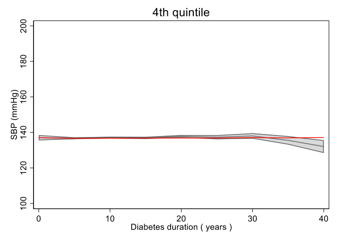
5	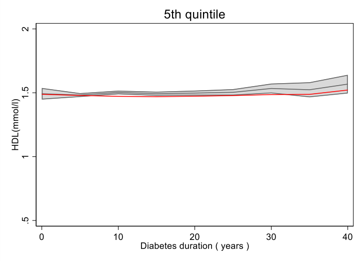	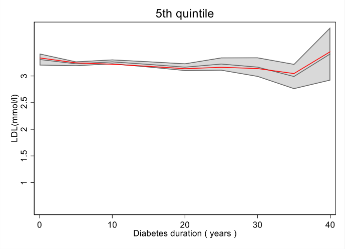	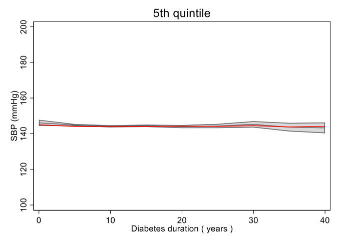

Figure A2. Observed values with 95% confidence intervals (grey area) and simulated* (red line) time paths by quintile of continuous risk factors (continued)

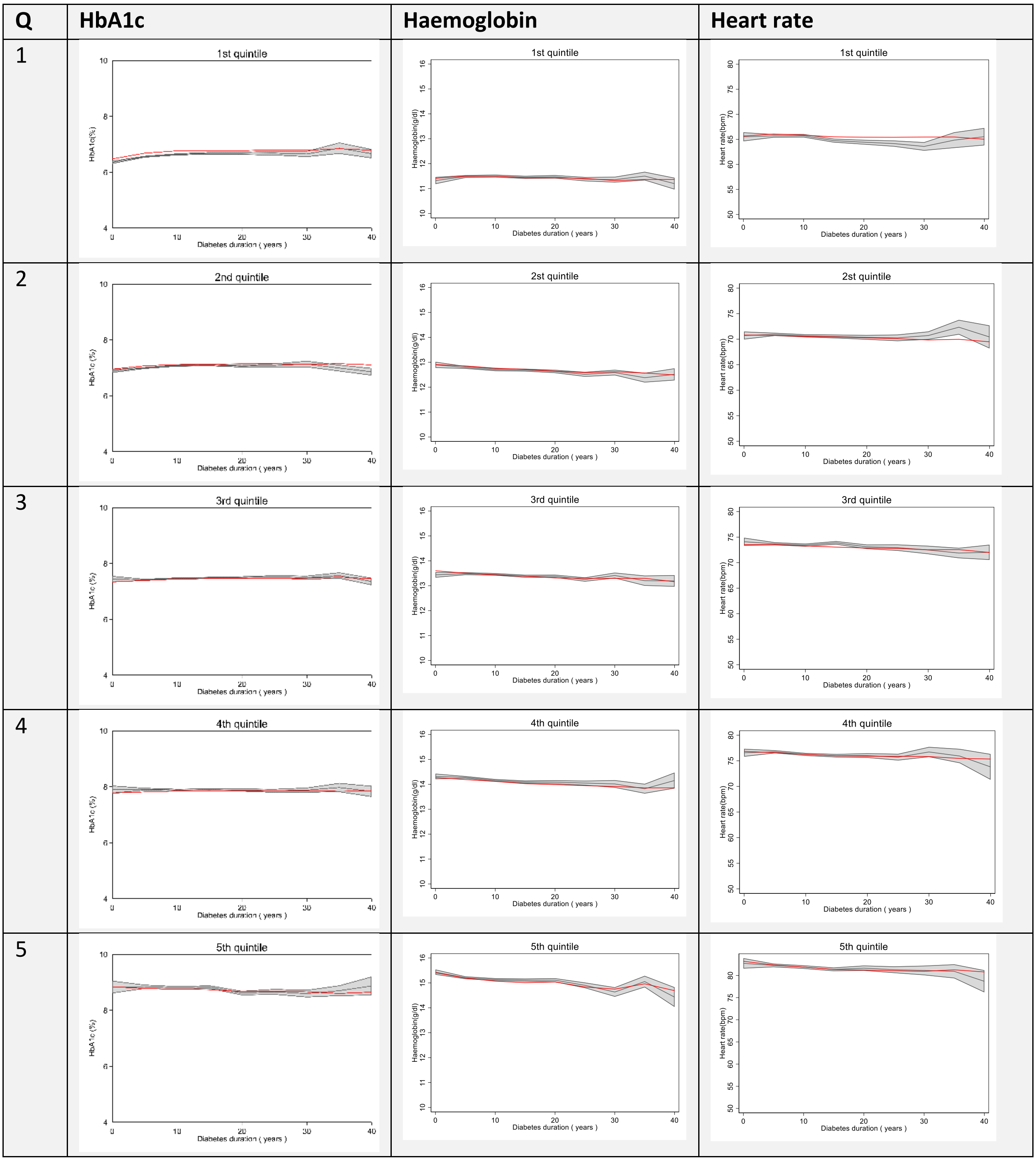

Figure A2. Observed values with 95% confidence intervals (grey area) and simulated* (red line) time paths by quintile of continuous risk factors (continued)

Q	BMI	eGFR
1	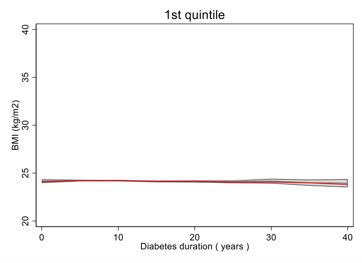	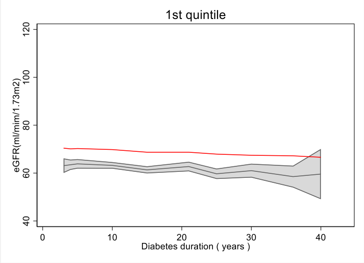
2	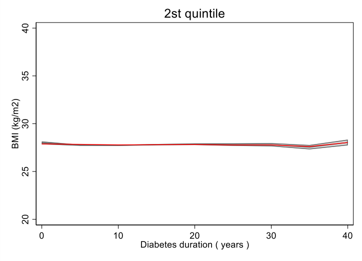	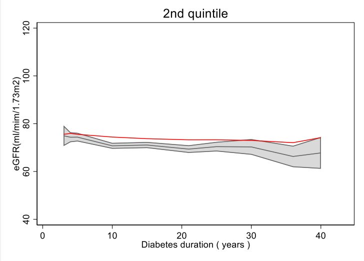
3	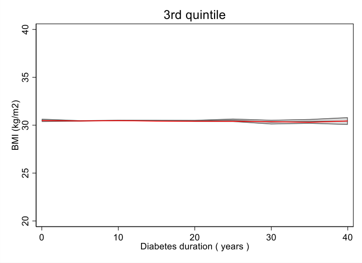	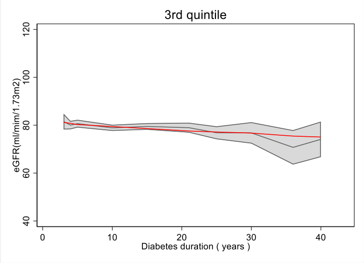
4	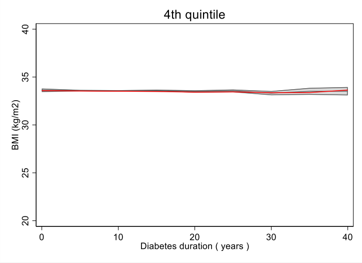	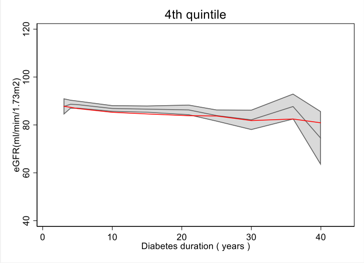
5	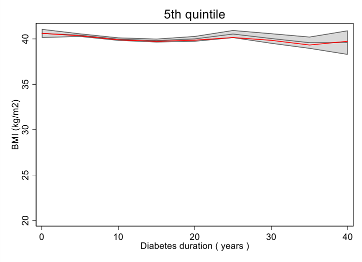	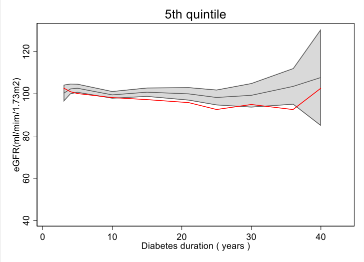

Figure A3. Observed values with 95% confidence intervals (grey area) and simulated time paths for continuous risk factors estimated in the current study (red line) and by Leal et al [6] (blue line), relative to randomisation.

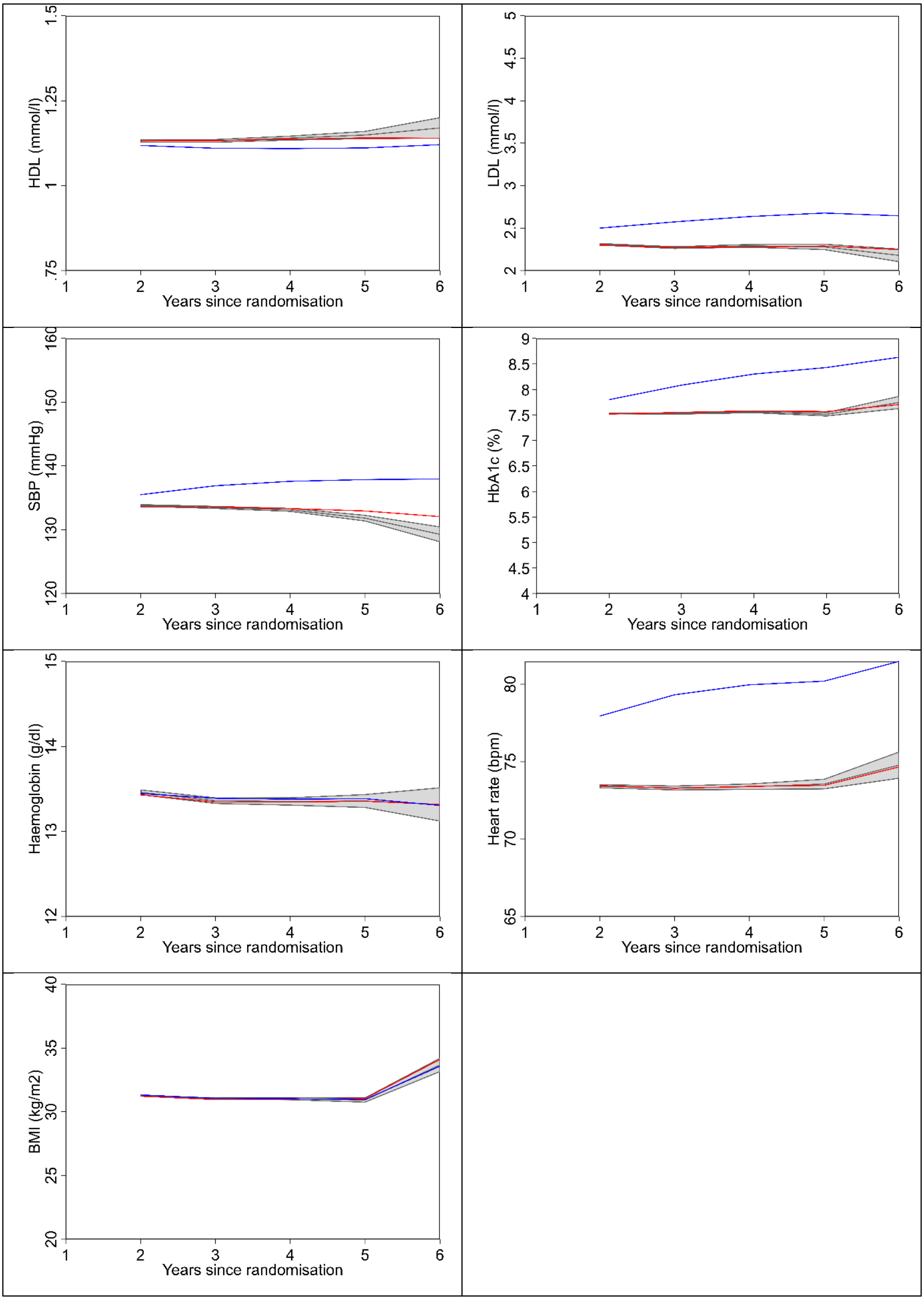

Abbreviations: BMI, body mass index (BMI); HbA_{1c}, glycated haemoglobin; HDL-C, high-density lipoprotein cholesterol; LDL-C, low-density lipoprotein cholesterol; SBP, systolic blood pressure.

Table A5. Coefficients for the models estimating annual risk factor values of continuous variables using solely EXSCEL data

VARIABLES	HDL	LDL	SBP	HbA1c	Haemoglobin	Heart rate	BMI
Value Y in previous year	0.187***	0.304***	0.257***	0.440***	0.325***	0.258***	0.766***
	(0.028)	(0.015)	(0.010)	(0.012)	(0.021)	(0.010)	(0.011)
First recorded value Y	0.578***	0.410***	0.384***	0.273***	0.443***	0.426***	0.211***
	(0.031)	(0.016)	(0.011)	(0.013)	(0.023)	(0.011)	(0.012)
ln(duration of diabetes)	0.004	-0.041***	-0.053	0.066***	-0.093***	-0.366***	-0.025
	(0.003)	(0.011)	(0.165)	(0.014)	(0.020)	(0.105)	(0.018)
age at baseline	0.001***	-0.003***	0.027***	-0.011***	-0.009***	-0.044***	-0.004***
	(0.000)	(0.001)	(0.010)	(0.001)	(0.001)	(0.007)	(0.001)
female	0.053***	0.086***	0.193	0.018	-0.186***	0.408***	0.017
	(0.005)	(0.012)	(0.179)	(0.015)	(0.024)	(0.112)	(0.020)
White	0.020***	0.036	0.294	-0.070**	-0.052	0.487**	0.061
	(0.007)	(0.025)	(0.343)	(0.033)	(0.056)	(0.199)	(0.038)
Black	0.050***	0.069**	1.107**	0.083	-0.271***	0.202	-0.044
	(0.011)	(0.034)	(0.529)	(0.051)	(0.068)	(0.315)	(0.055)
Asia	0.017*	-0.028	0.660	-0.068	-0.009	1.422***	-0.040
	(0.009)	(0.030)	(0.445)	(0.042)	(0.071)	(0.280)	(0.043)
Constant	0.156***	0.908***	46.211***	2.898***	4.015***	26.363***	0.898***
	(0.017)	(0.056)	(1.110)	(0.091)	(0.207)	(0.728)	(0.105)
Observations	20,323	19,164	27,081	24,963	14,303	26,902	26,651
Number of id	9,673	9,211	12,311	11,431	7,321	12,263	12,134

Robust standard errors in parentheses; *** p<0.01, ** p<0.05, * p<0.1

Table A6. Coefficients for the models estimating annual risk factor values of continuous variables using solely TECOS data

VARIABLES	HDL	LDL	SBP	HbA1c	Haemoglobin	Heart rate	BMI
Value Y in previous year	0.297***	0.274***	0.282***	0.516***	0.321***	0.303***	0.605***
	(0.021)	(0.018)	(0.008)	(0.013)	(0.023)	(0.009)	(0.037)
First recorded value Y	0.427***	0.354***	0.226***	0.153***	0.484***	0.277***	0.361***
	(0.025)	(0.018)	(0.008)	(0.013)	(0.025)	(0.010)	(0.037)
ln(duration of diabetes)	-0.007**	-0.042***	0.062	0.063***	-0.139***	-0.233**	0.014
	(0.003)	(0.010)	(0.180)	(0.010)	(0.024)	(0.107)	(0.019)
age at baseline	0.001***	-0.003***	0.026**	-0.009***	-0.009***	-0.046***	-0.012***
	(0.000)	(0.001)	(0.013)	(0.001)	(0.002)	(0.008)	(0.001)
female	0.054***	0.109***	0.558***	0.019	-0.210***	0.612***	0.067***
	(0.005)	(0.014)	(0.210)	(0.012)	(0.030)	(0.129)	(0.024)
White	0.017**	-0.060**	0.177	-0.112***	-0.067	-0.039	-0.045
	(0.007)	(0.030)	(0.423)	(0.026)	(0.051)	(0.240)	(0.042)
Black	0.035***	-0.046	0.039	0.004	-0.138*	0.516	-0.133*
	(0.013)	(0.045)	(0.765)	(0.045)	(0.081)	(0.460)	(0.074)
Asia	0.012	-0.064**	-1.054**	-0.056**	-0.079	1.558***	-0.159***
	(0.008)	(0.030)	(0.450)	(0.028)	(0.053)	(0.264)	(0.045)
Constant	0.215***	1.134***	63.672***	2.926***	3.525***	33.435***	1.710***
	(0.021)	(0.068)	(1.307)	(0.085)	(0.226)	(0.846)	(0.172)
Observations	17,469	16,390	27,709	26,490	8,354	27,026	27,234
Number of id	8,826	8,312	11,804	11,310	4,795	11,604	11,613

Robust standard errors in parentheses; *** p<0.01, ** p<0.05, * p<0.1

Figure A4. Cross-validation analyses comparing the performance of models fitted on EXSCEL data (Table A5) and models fitted on TECOS data (Table A6) against observed data from either EXSCEL or TECOS.

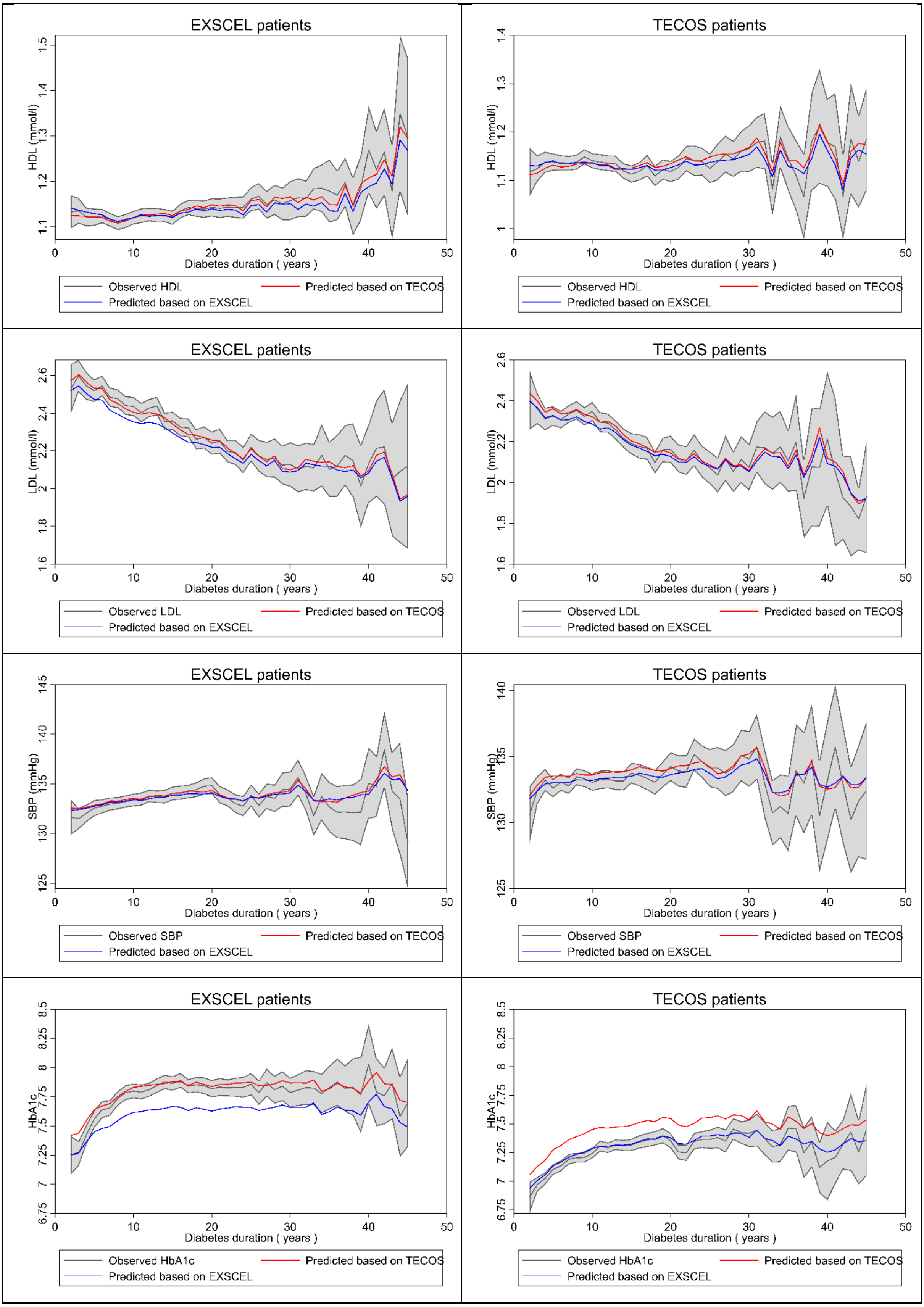

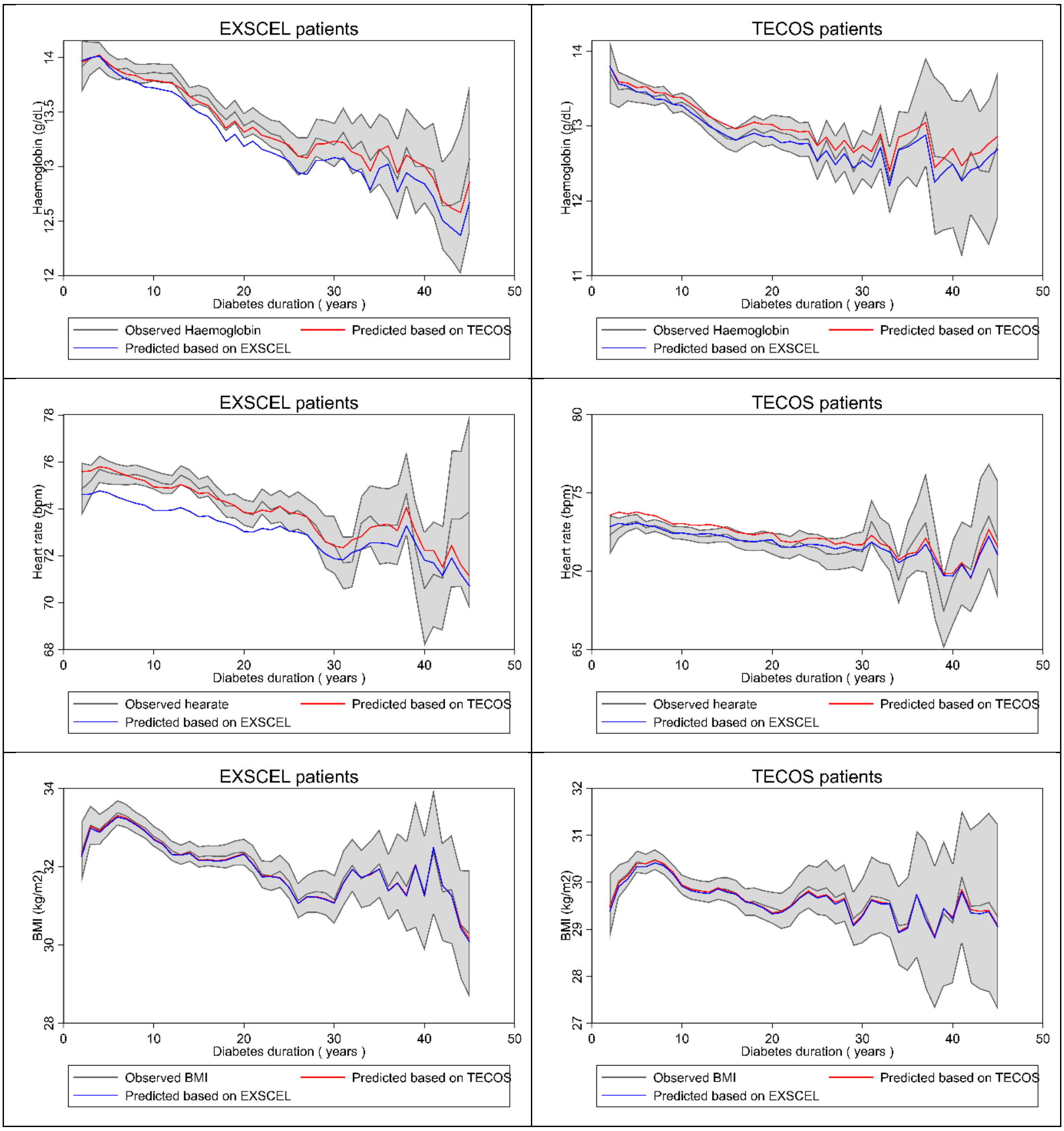

Abbreviations: BMI, body mass index (BMI); HbA_{1c}, glycated haemoglobin; HDL-C, high-density lipoprotein cholesterol; LDL-C, low-density lipoprotein cholesterol; SBP, systolic blood pressure.

Supplementary material 2: Instructions on how to use the coefficients to predict risk factors

Tables 1 and 2 in the manuscript provide coefficients for the estimation of the continuous and binary risk factors of an individual patient for each year of simulation, based on their predicted risk factor value in the previous year and their risk factor value at the start of the simulation. The values at the start of the simulation may represent the last recorded value in a randomised trial.

This supplementary material describes how these coefficients should be applied, using a hypothetical individual who had the risk factor values shown in Table A7 at the start of the simulation. The methods for applying these coefficients are similar to those for Leal et al 2021 [6].

Table A7. Risk factor values for hypothetical individual at the start of the simulation. These are based on the Mount Hood reference case simulation [7]

Risk factor	Value at start of simulation
Sex	Male
Ethnicity	White*
Smoker	No
Age, years	66
Duration of diabetes, years	8
Age at diagnosis	58
HbA1c, %	7.5
Height (m)	1.7
Weight (kg)	80.92
BMI, kg/m^2	28.69
LDL, mmol/l	3.0
HDL, mmol/l	1.3
SBP, mmHg	145
eGFR, ml/min/1.73m^2	70
Heart rate, bpm	79
Haemoglobin, g/dl	14
White blood cell count x10^9/l	7
PVD	No
Atrial fibrillation	No
Albuminuria	No

* Assumed for the purposes of this simulation: not specified in the Mount Hood reference.

Estimating predictions for continuous risk factors

As described in the Methods, continuous risk factors, such as HbA1c are predicted as

$$y_{it} = \phi_0 + \phi_1 y_{it-1} + \phi_2 \mathrm{y}_{\mathrm{i},0} + \phi_3 sex_i + \phi_4 ethnic_{ij} + \phi_5 age_i + \phi_6 \ln(duration\ of\ diabetes_{it})$$

where y_{it} is the value of risk factor for individual *i* in year *t*. $y_{i,t-1}$ is the previous year's risk factor value; $\mathrm{y}_{\mathrm{i},0}$ is the risk factor value at start of simulation[6] and $ethnic_{ij}$ is a series of dummy variables for ethnicity, with '1' indicating White for $ethnic_{i1}$, Black for $ethnic_{i2}$, or Asian (oriental, Indian or other) for $ethnic_{i1}$. The baseline ethnicity category is other (Hispanic, Aboriginal (Australia), Maori, Native Hawaiian, Pacific Islander, Indian (American) or Alaska Native). Finally, age_i is age at the start of the simulation.

We can insert the coefficient values from Table 1 to estimate predictions for HbA1c for the hypothetical individual shown in Table A7 12 months after the start of the simulation (9 years of duration of diabetes) as:

$$HbA1c_{Person1Year1} = 2.945 + 0.456 * 7.5 + 0.243 * 7.5 + 0 * 0.031 - 0.082 - 0.012 * 66 + 0.083 * \ln(8 + 1) = 7.496$$

In Year 2, we can insert the Year 1 HbA1c as the HbA1c last year and increase the duration of diabetes by 1 to give:

$$HbA1c_{Person1Year2} = 2.945 + 0.456 * \mathbf{7.496} + 0.243 * 7.5 + 0 * 0.031 - 0.082 - 0.012 * 66 + 0.083 * \ln(\mathbf{8} + \mathbf{2}) = 7.503$$

The same methods can be used to estimate predictions for other continuous risk factors using the coefficients in Table 1.

Estimating predictions for binary risk factors

Atrial fibrillation (AF), albuminuria (ALB), PVD and eGFR <60 ml/min/1.73m^2 were predicted based on Weibull proportional hazards models (Table 2). We assumed that once a patient has been diagnosed with one of these events, they will have it for the rest of their life. The unconditional probability of these events occurring in the interval *t* to *t+1* is:

$$1 - \exp(H(t|x_{jt}) - H(t + 1|x_{jt}))$$

Where $H(t|x_{jt})$ is the integrated cumulative hazard at time *t* (years since diagnosis of diabetes) defined for the Weibull regression as

$$H(t|x_{jt}) = \exp(\beta_0 + \beta_j x_{tj})t^{\gamma}$$

where x_{tj} is the vector of the covariates reported in Table A7 and β_j is the vector of their respective coefficients (Table 2).

Hence, the probability of atrial fibrillation (AF) 12 months after the start of the simulation for the individual outlined in Table A7 can be calculated as follows. First we estimate the cumulative hazards at time t and time t+1.

$$H_{AF}(t|x_{jt}) = \exp(\beta_0 + \beta_1 AgeAtRandomisation + \beta_2 Female + \beta_3 White + \beta_4 Black + \beta_5 BMIpreviousyear + \beta_6 HDLpreviousyear \quad)t^{\Gamma}$$

Hence for $t_1 = 9$ (year 1 of the simulation, where duration of diabetes is 9 years)

$$H_{AF}(t_1|x_{j1}) = \exp(-11.269 + 0.060 * 66 - 0.576 * 0 + 1.073 * 1 + 1.150 * 0 + 0.058 * 28.69 - 0.487 * 1.3) * (8 + 1)^{1.210} = 0.07836$$

And $t_2 = 10$ (year 2 of the simulation, where duration of diabetes is 10 years [assuming for simplicity that BMI and LDL have remained unchanged])

$$H_{AF}(t_2|x_{j1}) = \exp(-11.269 + 0.060 * 66 - 0.576 * 0 + 1.073 * 1 + 1.150 * 0 + 0.058 * 28.69 - 0.487 * 1.14) * (8 + 2)^{1.210} = 0.09623$$

Where Γ =1.210 i.e. exp(ln(Γ)) in Table 2 of the manuscript.

Second, we estimate the probability of AF in the first year of the simulation to be 1.8% as,

$$ProbabilityAF = 1 - \exp(0.07836 - 0.09623) = 0.01771$$

This probability is then compared against a random draw from a uniform (0,1) distribution and, if it is higher, the individual develops AF.

The same approach is used to predict the probability of albuminuria, PVD and eGFR <60 ml/min/1.73m^2 conditional on the Weibull model covariates reported in Table 2. Smoking at randomisation was a significant predictor of PVD (Table 1); when predicting PVD, a dummy variable indicating whether the patient smoked at the start of the model simulation can be used in place of a dummy indicating whether the patient smoked at randomisation.

Predicting eGFR

A two-step approach was used to predict eGFR for each year of the UKPDS-OM2 simulation. The first step comprised a proportional hazard Weibull survival model predicting the probability that eGFR is <60 ml/min/1.73m^2 for each year of simulation. As with the other survival equations, once an individual progresses to eGFR<60 they remain in this health state for the rest of the simulation. The second step uses one of two Tobit models to predict the patient's eGFR value (as a continuous variable) conditional on whether they were predicted to have eGFR above or below 60 ml/min/1.73m^2 in the first step.

The Weibull model and Tobit models described in the main manuscript were used to predict eGFR for each patient one year at a time. For each patient with eGFR>60 ml/min/1.73m^2 in the previous year, in each loop of the UKPDS-OM simulation, the predicted probability of the Weibull model is compared with a random number to determine whether the patient progressed to eGFR<60 ml/min/1.73m^2 that year. Then, conditional on progression or not to eGFR<60

ml/min/1.73m^2, one of the Tobit models is used to predict that patient's eGFR value. For patients with eGFR<60 ml/min/1.73m^2 in the previous year, the patient is assumed to remain in the eGFR<60 ml/min/1.73m^2 health state and the Tobit model for eGFR<60 ml/min/1.73m^2 is used to predict eGFR for that year.

For the first step, the instructions for binary risk factors in the previous section can be followed to estimate the predicted probability that eGFR is <60 ml/min/1.73m^2 and determine whether the patient progresses to eGFR<60 ml/min/1.73m^2 that year (by comparing predicted probability against random draw from uniform distribution (0,1)).

If patient i is determined to have eGFR below 60 ml/min/1.73m^2, the conditional expected eGFR value is predicted as:

$$E(eGFR_i|0 < eGFR_i^* < 60) = \beta' x_i - \sigma\frac{\phi_{1i} - \phi_{2i}}{\Phi_{1i} - \Phi_{2i}}$$

Where: $\Phi_{1i} = \Phi(\frac{0-\beta' x_i}{\sigma})$ is the cumulative distribution function for a standard normal distribution using the lower limit (0 ml/min/1.73m^2); $\Phi_{2i} = \Phi(\frac{60-\beta' x_i}{\sigma})$ is the cumulative distribution function for a standard normal distribution using the upper limit (60 ml/min/1.73m^2); ϕ_{1i} and ϕ_{2i} are the corresponding density functions for the standard normal distribution; and σ is the sigma (standard error of the forecast) given in Table 2 (11.888).

$\beta' x_i$ is the linear predictor:

$$\beta' x_i = 36.058 + (-0.100) * ageatrandomisation - 0.025 * SBP + 0.232 * eGFRpreviousyear + 0.459 * eGFRatbaseline + (-1.335) * \ln(durationdiabetes)$$

For instance, for the hypothetical individual in Table A7 who was age 66 with eGFR of 70 ml/min/1.73m^2 and SBP of 145 mmHg at the start of the simulation, the linear predictor $\beta' x_i$ at the end of the first year of simulation (nine years after diagnosis of diabetes) can be calculated as follows if the first step predicts eGFR <60 ml/min/1.73m^2:

$$\beta' x_i = 36.058 + (-0.100) * 66 - 0.025 * 145 + 0.232 * 70 + 0.459 * 70 + (-1.335) * \ln(8 + 1\ years) = 71.2697$$

$\Phi_{1i} = \Phi\left(\frac{0-(71.2697)}{11.888}\right) = \Phi(-5.995) = 1.017 \times 10^{-9}$ and $\phi_{1i} = \phi(-5.995) = 6.257 \times 10^{-9}$

$\Phi_{2i} = \Phi\left(\frac{60-(71.2697)}{11.888}\right) = \Phi(-0.948) = 0.1716$ and $\phi_{2i} = \phi(-0.948) = 0.2545$

This patient will also be assumed to continue to have eGFR<60 ml/min/1.73m^2 for the rest of their life.

Then,

$E(eGFR_i|0 < eGFR_i^* < 60) = \beta' x_i - \sigma\frac{\phi_{1i}-\phi_{2i}}{\Phi_{1i}-\Phi_{2i}} = 71.2697 - 11.888 * \frac{6.257\times10^{-9}-0.2545}{1.017\times10^{-9}-0.1716} =$ 53.632 ml/min/1.73m^2

If patient i is determined to have eGFR above 60 ml/min/1.73m^2 in loop L, the conditional eGFR value is predicted as:

$$E(eGFR_i|eGFR_i^* \geq 60) = \beta' x_i + \sigma \frac{\phi_{1i}}{1 - \Phi_{1i}}$$

Where: $\Phi_{1i} = \Phi(\frac{60-\beta' x_i}{\sigma})$ is the cumulative distribution function for a standard normal distribution using the lower limit (60 ml/min/1.73m^2); ϕ_{1i} is the corresponding density functions for the standard normal distribution; σ is the sigma (standard error of the forecast) given in Table 2 (13.839).

For the same hypothetical individual in Table A7 who was age 66 with eGFR of 70 ml/min/1.73m^2 and SBP of 145 mmHg at the start of the simulation, the linear predictor $\beta' x_i$ at the end of the first year of simulation (nine years after diagnosis of diabetes) can be calculated as follows if the first step predicts eGFR ≥60 ml/min/1.73m^2 in loop L:

$\beta' x_i = 36.317 + (-0.230) * 66 - 0.011 * 145 + 0.446 * 70 + 0.303 * 70 +$
$(-0.753) * \ln(8 + 1\ years) = 70.3175$

$\Phi_{1i} = \Phi\left(\frac{60-(70.3175\ )}{13.839}\right) = \Phi(-0.746) = 0.228$ and $\phi_{1i} = \phi(-0.746) = 0.302$

Hence, $E(eGFR_i) = \beta' x_i + \sigma \frac{\phi_{1i}}{1-\Phi_{1i}} = 70.3175\ + 13.839 * \frac{0.302}{1-0.228} = 75.73$ ml/min/1.73m^2

If the patient did not progress to eGFR <60 ml/min/1.73m^2 in year 1, the eGFR predicted for that patient for year 1 in this loop was then used to predict the probability that this patient progressed to eGFR <60 ml/min/1.73m^2 the following year for the same loop. The eGFR for year 1 was also used to predict the exact eGFR value in the relevant Tobit model conditional on the prediction of the Weibull survival model. This was repeated for subsequent years for this patient in this loop and then repeated for multiple loops for the same patient, and then for other patients.

Notes regarding all time paths

Coefficients to more decimal places and those with an alternative ethnicity coding (which uses white as the baseline category, rather than other) are available from the corresponding author on request. A set of 5000 bootstrap estimates (with replacement) of all regression coefficients using this alternative ethnicity coding are also available on request, which could be used to propagate uncertainty around the risk that the trajectory equations within a model, such as UKPDS-OM2.

Since neither EXSCEL nor TECOS collected data on white blood cell count, or post-baseline smoking, the equations estimated by Leal et al could be used to project white blood cell count and smoking [6].

EXSCEL and TECOS were designed as glycaemic equipoise studies, i.e. “usual care physicians [were] encouraged to follow guidelines for care based on local and institutional practice

patterns and any relevant published practice guidelines" [2]. This differs considerably from previous diabetes trials where individuals were prescribed a placebo without usual care. See, for example, Bethel 2020 [8]. Both trials had a highly pragmatic design with very few restrictions on concomitant treatment and compared usual care plus study drug against usual care plus placebo. Hence, data from these trials are likely to be valid for contemporary populations that are similar to those in the trials.

In addition to the study medication evaluated in EXSCEL and TECOS, there have been many other new drugs and many changes to clinical management and secular trends since the UKPDS era (1977-2002) that will affect risk factor time paths. For example, HbA1c may be affected by increased use of metformin, lower blood glucose targets, patient education, introduction of long-acting insulins, glitazones, as well as the newer drugs (e.g. empagliflozin) that were introduced during EXSCEL and TECOS follow up. LDL will be affected by the introduction of statins and change of guidance recommending lower LDL targets, higher intensity statin regimens and use in a wider range of patients. People diagnosed with diabetes in 1977-1997 are also a different generation from most of those participating in EXSCEL and TECOS and may have been less active in old age and be more likely to have smoked.

For each patient, it is likely that any change in medication (whether this is exenatide/sitagliptin, or concomitant drug) will produce a step change in risk factors in the first 6-12 months after changing medication. While patients are on stable treatment, the change in risk factors is likely to be relatively stable but there may be changes due to concomitant medication, lifestyle factors and increasing duration of diabetes. For example, an individual EXSCEL study participant may initially receive exenatide plus metformin and have a gradual increase in HbA1c over time. If their HbA1c exceeds a target level two years later, their clinician may decide to add insulin, resulting in a reduction in HbA1c. After that fall, HbA1c levels may then gradually rise and their clinician may increase the insulin dose accordingly, resulting in another rapid fall and then a gradual rise. Other patients may stop medications (e.g. due to patient preference, hypoglycaemia or adverse events). However, many patients may have no change in glucose-lowering medication during the study period and may have relatively stable HbA1c. Across our cohorts, there will be patients starting or stopping many different medications at different times. Our time path equations aim to predict the average risk factor trend over time given the current package of interventions (and controlling for demographics and past risk factor levels).

Users of diabetes simulation models, such as UKPDS-OM2, will typically use our risk factor time paths to model how risk factors change over time after the effect of study interventions has been applied. For example, researchers may do a network meta-analysis of 48-week trials to estimate the reduction in HbA1c during the initial year of treatment. They may reduce HbA1c in year 1 of simulation based on the meta-analysis treatment effects and use our risk factor time paths to model how HbA1c changes over time from year 2 onwards. Researchers evaluating treat-to-target regimens may also assume that patients intensify treatment when our time path equations predict that patients' HbA1c has risen above a target level and may then apply an initial treatment effect, followed by a further period extrapolated using our time path equations.

Supplementary material 3: Methods for QALY gains using current and previous risk equations extrapolated using UKPDS-OM2

The impact of different risk factor trajectories on QALYs was by extrapolating risk factors for 2579 participants randomised to placebo in either EXSCEL or TECOS who had complete data on all risk factors at baseline.

For each participant, we took the pre-randomisation values of each risk factor and extrapolated using either the risk factor trajectories estimated in this paper, or those estimated by Leal et al. [6].

Since neither trial measured white blood cell count, baseline white blood cell count was imputed using a published algorithm estimated using the UKPDS trial dataset [9]. Post-baseline values for white blood cell count and for smoking (where there was no data post-baseline) were estimated from the baseline value using the trajectories estimated by Leal et al. [6]. Continuous risk factors were extrapolated in Stata version 17.0 and entered into the model directly as fixed values.

A bespoke version of UKPDS-OM version 2.2 was used, which incorporated risk factor time paths using the equations of Leal et al. [6] or those of the current paper. As described in Supplementary material 2, for binary risk factors (PVD, albuminuria, atrial fibrillation and smoking) and eGFR, accurate estimation of lifetime QALYs requires Monte Carlo simulation. For PVD, albuminuria, atrial fibrillation and eGFR <60 ml/min/173m^2, the survival models predict the rate at which patients will develop risk factors, whereas the UKPDS-OM requires binary inputs for whether the patient has or has not developed that risk factor at each time point. Similarly, the logistic regression for smoking reported by Leal et al [6] predicts whether patients are current smokers or not. In each loop of the model, the hazard (or log-odds) for developing each risk factor in the next year is estimated and this hazard (or log-odds) is compared against random numbers to decide whether or not the patient develops that risk factor that year. Outcomes for the following year are estimated conditional on whether the patient developed it previous year. This approach ensures that the patient will develop each risk factor in the correct proportion of loops and ensures that the mean QALYs for each patient reflect that patient's true risk. By contrast, other methods, such as the highest probability approach, would introduce bias [10].

Results were extrapolated for 70 years using 100,000 loops. No bootstraps were used since the aim was to compare point estimates. The default utility values, estimated from the UKPDS sample [11], were used to estimate QALYs. No discounting was applied.

Supplementary material 4: Methods and results of reference simulation

The 12 reference simulations set out in https://www.mthooddiabeteschallenge.com/registry were run using the same bespoke version of UKPDS-OM version 2.2. The simulation engine and QALY calculations in the model we used is identical to that of the publically available models of version 2.0 and 2.2 but enables extrapolation of risk factors using the time path equations presented in the current paper as well as those of Leal et al. [6].

In line with the original reference simulation for UKPDS-OM version 2.0, we assumed patients were white and 1.7 m tall. We ran 50 million loops and did not consider parameter uncertainty. Following the methods for the Mount Hood registry, no increments were applied at baseline (only from year 1 onwards). We extrapolated the baseline outcomes for the control patients using each of the three methods and then applied the increments to year 1 onwards, relative to the values for the control patient in that year (Tables A8 and A9).

Predicted risk factors from this simulation are plotted for the control patients in Figure A5.

Table A8. Baseline inputs for reference simulation. The columns for IHD, Heart failure, Amputation, Blindness, Renal failure, Stroke, MI and ulcer have missing values for all patients (indicating no history of these events at start of simulation).

		Demographic characteristics						Risk factor values at start of simulation												Discounting start year
ID	Group	Ethnicity	Gender	Age now	Duration of diabetes	Weight	Height	AF	PVD	Current smoker	Album-inuria	HDL	LDL	Systolic BP	HbA1c	Heart rate	WBC	Haem-oglobin	eGFR	
Control male	1	1	M	66	8	80.92	1.7	N	N	N	N	1.3	3	145	7.5	79	7	14	70	0
Control female	2	1	F	66	8	80.92	1.7	N	N	N	N	1.3	3	145	7.5	79	7	14	70	0
0.5%-point reduction in HbA1c Male	3	1	M	66	8	80.92	1.7	N	N	N	N	1.3	3	145	7.5	79	7	14	70	0
0.5%-point reduction in HbA1c Female	4	1	F	66	8	80.92	1.7	N	N	N	N	1.3	3	145	7.5	79	7	14	70	0
10mm Hg reduction in SBP male	5	1	M	66	8	80.92	1.7	N	N	N	N	1.3	3	145	7.5	79	7	14	70	0
10mm Hg reduction in SBP female	6	1	F	66	8	80.92	1.7	N	N	N	N	1.3	3	145	7.5	79	7	14	70	0
0.5 mmol/l reduction in LDL male	7	1	M	66	8	80.92	1.7	N	N	N	N	1.3	3	145	7.5	79	7	14	70	0
0.5 mmol/l reduction in LDL female	8	1	F	66	8	80.92	1.7	N	N	N	N	1.3	3	145	7.5	79	7	14	70	0
1-unit reduction in BMI (kg/m2) male	9	1	M	66	8	80.92	1.7	N	N	N	N	1.3	3	145	7.5	79	7	14	70	0
1-unit reduction in BMI (kg/m2) female	10	1	F	66	8	80.92	1.7	N	N	N	N	1.3	3	145	7.5	79	7	14	70	0
All interventions combined male	11	1	M	66	8	80.92	1.7	N	N	N	N	1.3	3	145	7.5	79	7	14	70	0
All interventions combined female	12	1	F	66	8	80.92	1.7	N	N	N	N	1.3	3	145	7.5	79	7	14	70	0

Table A9. Example of BMI inputs for reference simulation

	BMI trajectory LOCF					BMI trajectories from current paper				
**ID**	**Year 1**	**Year 2**	**Year 3**	**Year 4**	**Year 5**	**Year 1**	**Year 2**	**Year 3**	**Year 4**	**Year 5**
Control male	80.92	80.92	80.92	80.92	80.92	80.80	80.71	80.65	80.61	80.59
Control female	80.92	80.92	80.92	80.92	80.92	80.91	80.90	80.90	80.89	80.89
0.5%-point reduction in HbA1c Male	80.92	80.92	80.92	80.92	80.92	80.80	80.71	80.65	80.61	80.59
0.5%-point reduction in HbA1c Female	80.92	80.92	80.92	80.92	80.92	80.91	80.90	80.90	80.89	80.89
10mm Hg reduction in SBP male	80.92	80.92	80.92	80.92	80.92	80.80	80.71	80.65	80.61	80.59
10mm Hg reduction in SBP female	80.92	80.92	80.92	80.92	80.92	80.91	80.90	80.90	80.89	80.89
0.5 mmol/l reduction in LDL male	80.92	80.92	80.92	80.92	80.92	80.80	80.71	80.65	80.61	80.59
0.5 mmol/l reduction in LDL female	80.92	80.92	80.92	80.92	80.92	80.91	80.90	80.90	80.89	80.89
1-unit reduction in BMI (kg/m2) male	78.03	78.03	78.03	78.03	78.03	77.91	77.82	77.76	77.72	77.70
1-unit reduction in BMI (kg/m2) female	78.03	78.03	78.03	78.03	78.03	78.02	78.01	78.01	78.00	78.00
All interventions combined male	78.03	78.03	78.03	78.03	78.03	77.91	77.82	77.76	77.72	77.70
All interventions combined female	78.03	78.03	78.03	78.03	78.03	78.02	78.01	78.01	78.00	78.00

Table A10. Results of reference simulation: QALYs for each hypothetical individual defined in the Mount Hood reference. The results for the model we used (version 2.2 Global) and the 2.0 release version are negligible and result purely from Monte Carlo error.

Model	Version	Extrapolation	Sex	Control	0.5% red. HbA1c	10mmHg in SBP	0.5mmol/l red. LDL	1-unit red BMI	All combined	Absolute QALY gain for all combined vs control	Date of simulation
UKPDS-OM	2.2 global 2023	Trajectories from current paper*	Male	11.97	12.07	12.15	12.19	12.00	12.47	0.50	12/09/2023
UKPDS-OM	2.2 global 2023		Female	13.47	13.56	13.64	13.59	13.50	13.86	0.39	12/09/2023
UKPDS-OM	2.2 global 2023	Leal et al [6] trajectories	Male	10.55	10.70	10.84	10.84	10.59	11.30	0.75	2/10/2023
UKPDS-OM	2.2 global 2023		Female	11.87	12.01	12.17	12.06	11.92	12.53	0.66	2/10/2023
UKPDS-OM	2.2 global 2023	LOCF	Male	11.90	11.99	12.09	12.13	11.92	12.41	0.51	13/09/2023
UKPDS-OM	2.2 global 2023		Female	13.56	13.64	13.74	13.69	13.59	13.95	0.39	13/09/2023
UKPDS-OM	2.0 release version	LOCF	Male	11.90	11.99	12.08	12.13	11.92	12.41	0.51	05/10/2018
UKPDS-OM	2.0 release version		Female	13.57	13.64	13.74	13.69	13.59	13.95	0.39	05/10/2018

LOCF, last observation carried forward (assuming no change in risk factors over 40 years, other than the increment applied at year one).
* Trajectories for smoking and WBC taken from Leal et al. [6].

Figure A5. Predicted risk factors for male (blue lines) and female (red lines) control patients defined by the Mount Hood Registry extrapolated over a lifetime using the time paths estimated in the current study. Values were estimated using a beta version of UKPDS-OM2; predicted cases of binary endpoints allow for death as a competing risk.

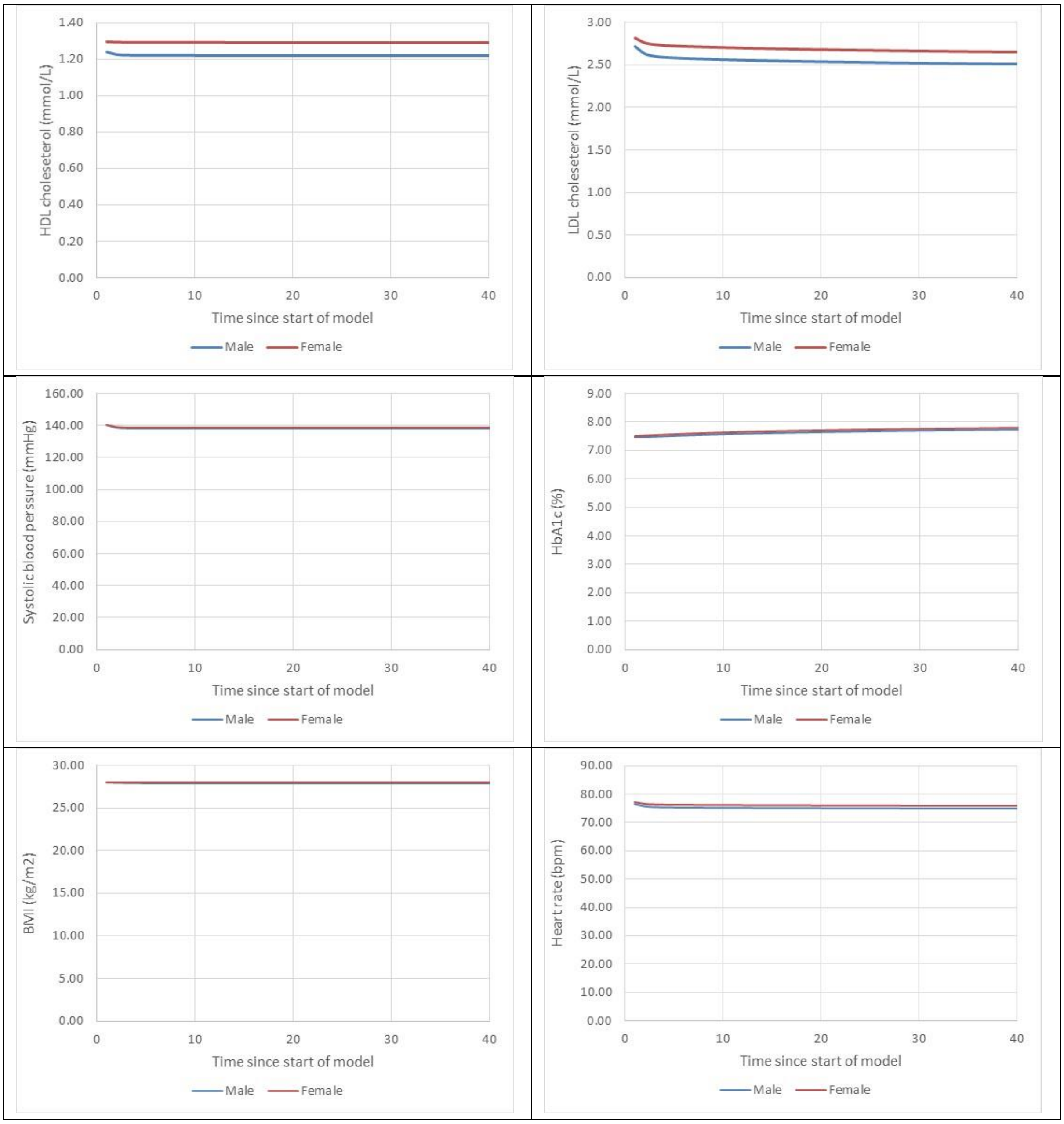

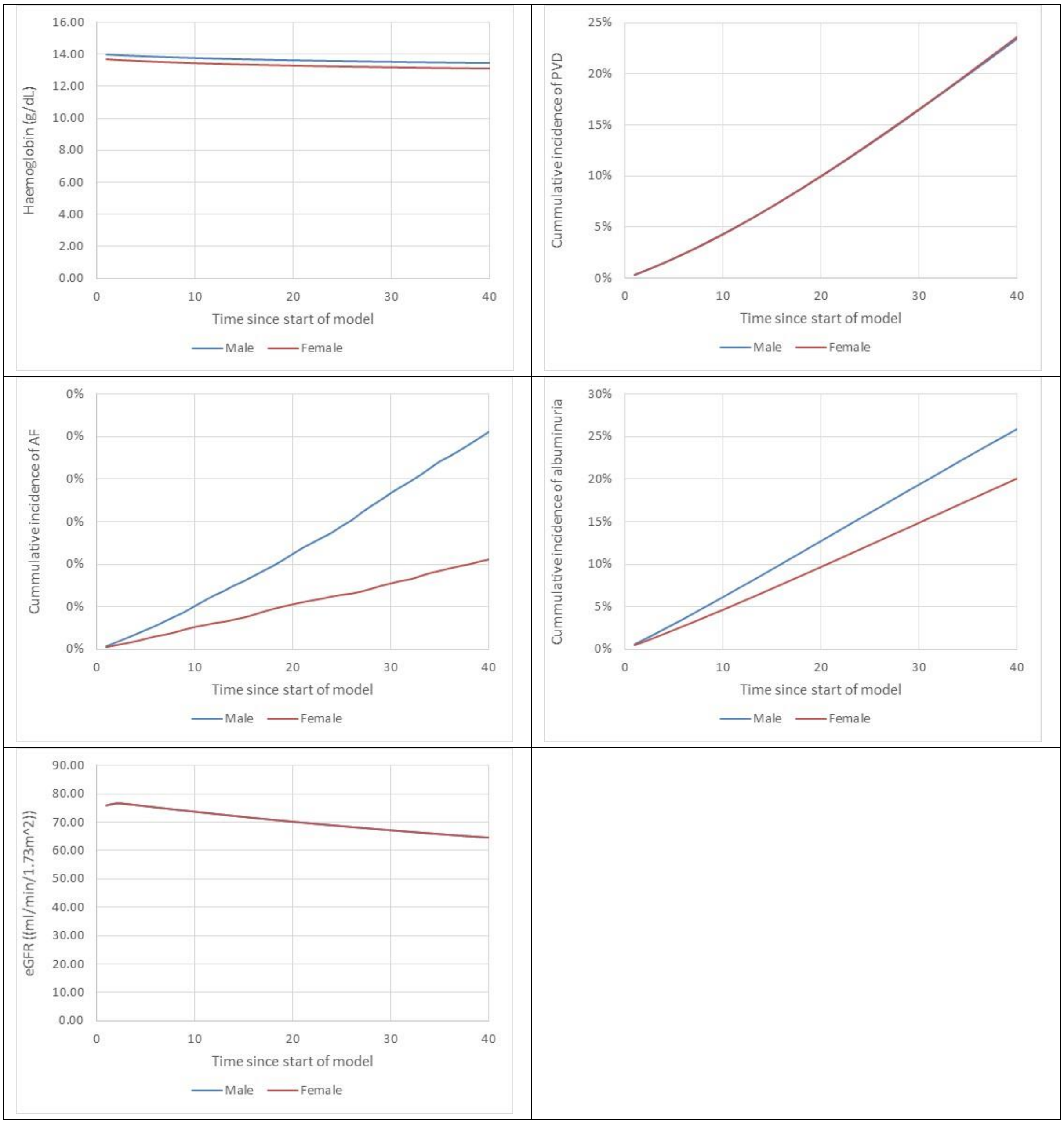

Abbreviations: AF, atrial fibrillation; BMI, body mass index (BMI); eGFR, estimated glomerular filtration rate; HbA_{1c}, glycated haemoglobin; HDL-C, high-density lipoprotein cholesterol; LDL-C, low-density lipoprotein cholesterol; PVD, peripheral vascular disease; SBP, systolic blood pressure.

Supplementary material 5: Evaluating impact of randomised treatment group on time paths ≥12 months after start of treatment

Post hoc analyses were conducted to test whether randomised treatment allocation affected continuous risk factor time paths. These were conducted by replicating the study-specific models shown in Tables A5 and A6 with the addition of a dummy indicating treatment allocation.

Treatment allocation was statistically significant ($p<0.05$) for only three combinations of study/risk factor: exenatide allocation had a significant effect on time paths for systolic blood pressure ($p=0.012$) and heart rate ($p=0.014$) while sitagliptin affected time paths for HbA1c ($p=0.001$). However, it should be noted that this is a post hoc analysis including 14 statistical tests, so has a high risk of a type 1 error.

For the three risk factor/study combinations where a significant difference was observed, we present coefficients separately for each treatment group (Supplementary material Table A11).

Table A11. Coefficients for the models estimating annual risk factor values of continuous variables separately for individual study arms. For brevity, the table shows only those combinations of study and risk factor for which treatment allocation had a statistically significant effect (p<0.05) when added to the regression.

VARIABLES	SBP	SBP	Heart rate	Heart rate	HbA1c	HbA1c
Trial	EXSCEL	EXSCEL	EXSCEL	EXSCEL	TECOS	TECOS
Treatment allocation	Exenatide	Placebo	Exenatide	Placebo	Sitagliptin	Placebo
Value Y in previous year	0.241** (0.013)	0.273** (0.014)	0.275** (0.015)	0.236** (0.014)	0.535** (0.018)	0.494** (0.019)
First recorded value Y	0.410** (0.015)	0.357** (0.015)	0.384** (0.016)	0.468** (0.016)	0.146** (0.017)	0.157** (0.019)
ln(duration of diabetes)	0.089 (0.227)	-0.209 (0.238)	-0.303** (0.149)	-0.411** (0.147)	0.061** (0.014)	0.066** (0.015)
Age at baseline	0.018 (0.014)	0.035** (0.015)	-0.045** (0.009)	-0.043** (0.009)	-0.010** (0.001)	-0.008** (0.001)
Female	0.134 (0.251)	0.233 (0.253)	0.319** (0.156)	0.477** (0.162)	0.023 (0.017)	0.015 (0.018)
White	0.480 (0.491)	0.092 (0.48)	0.437 (0.288)	0.536** (0.273)	-0.110** (0.036)	-0.113** (0.039)
Black	0.568 (0.762)	1.679** (0.732)	0.249 (0.427)	0.163 (0.468)	-0.014 (0.066)	0.021 (0.061)
Asian	0.209 (0.624)	1.066 (0.632)	1.333** (0.414)	1.567** (0.376)	-0.093** (0.038)	-0.012 (0.041)
Constant	44.754** (1.604)	47.886** (1.546)	28.375** (1.033)	24.726** (1.06)	2.906** (0.116)	3.002** (0.126)
Sigma_u	5.510	5.097	3.337	3.656	0.222	0.240
Sigma_e	9.660	10.028	6.005	5.821	0.601	0.626
rho	0.245	0.205	0.236	0.283	0.120	0.128
Observations	13,791	13,290	13,693	13,209	13,355	13,135
Number of id	6,188	6,123	6,156	6,107	5,683	5,627

Robust standard errors in parentheses** p<0.05

References for supplementary material

1. Holman RR, Bethel MA, George J, Sourij H, Doran Z, Keenan J, et al. Rationale and design of the EXenatide Study of Cardiovascular Event Lowering (EXSCEL) trial. Am Heart J. 2016;174:103-10. https://doi.org/10.1016/j.ahj.2015.12.009.
2. Holman RR, Bethel MA, Mentz RJ, Thompson VP, Lokhnygina Y, Buse JB, et al. Effects of Once-Weekly Exenatide on Cardiovascular Outcomes in Type 2 Diabetes. N Engl J Med. 2017;377:1228-39. https://doi.org/10.1056/NEJMoa1612917.
3. Green JB, Bethel MA, Paul SK, Ring A, Kaufman KD, Shapiro DR, et al. Rationale, design, and organization of a randomized, controlled Trial Evaluating Cardiovascular Outcomes with Sitagliptin (TECOS) in patients with type 2 diabetes and established cardiovascular disease. American Heart Journal. 2013;166:983-9. e7. https://doi.org/10.1016/j.ahj.2013.09.003.
4. Mentz RJ, Bethel MA, Gustavson S, Thompson VP, Pagidipati NJ, Buse JB, et al. Baseline characteristics of patients enrolled in the Exenatide Study of Cardiovascular Event Lowering (EXSCEL). Am Heart J. 2017;187:1-9. https://doi.org/10.1016/j.ahj.2017.02.005.
5. Bethel MA, Green JB, Milton J, Tajar A, Engel SS, Califf RM, et al. Regional, age and sex differences in baseline characteristics of patients enrolled in the Trial Evaluating Cardiovascular Outcomes with Sitagliptin (TECOS). Diabetes Obes Metab. 2015;17:395-402. https://doi.org/10.1111/dom.12441.
6. Leal J, Alva M, Gregory V, Hayes A, Mihaylova B, Gray AM, et al. Estimating risk factor progression equations for the UKPDS Outcomes Model 2 (UKPDS 90). Diabet Med. 2021:e14656. https://doi.org/10.1111/dme.14656.
7. Kent S, Becker F, Feenstra T, Tran-Duy A, Schlackow I, Tew M, et al. The Challenge of Transparency and Validation in Health Economic Decision Modelling: A View from Mount Hood. Pharmacoeconomics. 2019;37:1305-12. https://doi.org/10.1007/s40273-019-00825-1.
8. Bethel MA, Stevens SR, Buse JB, Choi J, Gustavson SM, Iqbal N, et al. Exploring the Possible Impact of Unbalanced Open-Label Drop-In of Glucose-Lowering Medications on EXSCEL Outcomes. Circulation. 2020;141:1360-70. https://doi.org/10.1161/CIRCULATIONAHA.119.043353.
9. Pagano E, Konings SRA, Di Cuonzo D, Rosato R, Bruno G, van der Heijden AA, et al. Prediction of mortality and major cardiovascular complications in type 2 diabetes: External validation of UK Prospective Diabetes Study outcomes model version 2 in two European observational cohorts. Diabetes Obes Metab. 2021;23:1084-91. https://doi.org/10.1111/dom.14311.
10. Ramos-Goñi J, Rivero-Arias O, Dakin H. Response mapping to translate health outcomes into the generic health-related quality of life instrument EQ-5D: Introducing the mrs2eq and oks2eq commands. Stata Journal. 2013;13:474-91.
11. Alva M, Gray A, Mihaylova B, Clarke P. The effect of diabetes complications on health-related quality of life: the importance of longitudinal data to address patient heterogeneity. Health Econ. 2014;23:487-500. https://doi.org/10.1002/hec.2930.
